# Synthesis of colicin Ia neoglycoproteins: tools towards glyco-engineering of bacterial cell surfaces†

**DOI:** 10.1039/d4ra04774e

**Published:** 2024-09-13

**Authors:** Natasha E. Hatton, Laurence G. Wilson, Christoph G. Baumann, Martin A. Fascione

**Affiliations:** a Department of Chemistry, University of York York YO10 5DD UK martin.fascione@york.ac.uk; b School of Engineering, Physics and Technology, University of York York YO10 5DD UK; c Department of Biology, University of York York YO10 5DD UK christoph.baumann@york.ac.uk

## Abstract

Colicins are antimicrobial proteins produced by certain strains of *Escherichia coli* that function as offensive weapons against closely-related competitor strains. Their bactericidal properties and narrow bacterial targeting range has made them of therapeutic interest. Furthermore, the applications of engineered non-bactericidal colicins are of interest as a cell surface-directed protein anchor for decorating *E. coli* with biomolecules. We previously demonstrated that an engineered non-bacteriocidal colicin E9 could be used to label bacterial cells with multiple biomolecules including glycans. Herein we extend our approach to colicin Ia, constructing mannose-presenting colicin la neoglycoproteins, through N-terminal organocatalyst-mediated protein aldol ligation (OPAL), or maleimide ligation targeting an internal cysteine. This work further highlights the potential utility of engineered colicins for non-genetic glyco-engineering of the *E. coli* cell surface.

## Abstract 2

Herein we demonstrate organocatalyst-mediated protein aldol ligation (OPAL) modification of the bacteriocidin colicin Ia to afford mannose-presenting neoglycoproteins with potential utility for non-genetic glyco-engineering of *E. coli* cell surfaces.

## Graphical abstract

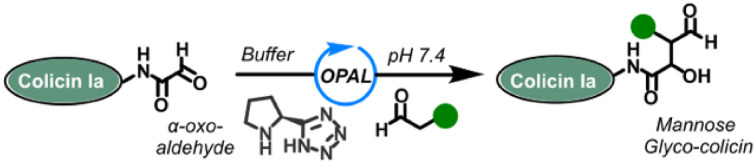

## 1. Introduction

Colicins are small proteins released by *Escherichia coli* strains which carry the colicinogenic plasmid.^1^ The released colicins can function as offensive weapons against competing *E. coli* strains not carrying the plasmid, and the immunity protein it encodes, by exerting their cytotoxic effect.^2,3^ Although the therapeutic applications of colicins (and colicin-like bacteriocins) are yet to be fully explored, multiple studies have demonstrated their antimicrobial potential.^4–6^ Colicins are classified into two groups (group A and group B); both groups are encoded on an extrachromosomal plasmid and the respective antimicrobial proteins are released into the surrounding medium. Group A colicins are translocated into the target *E. coli* cell using the Tol system, and include colicin A and colicins E1–E9.^3^ Group B colicins are translocated into the target *E. coli* cell using the Ton system, and include colicins B, D, Ia, Ib, M, 5 and 10.^3^

Colicins are of therapeutic interest due to both their narrow bacterial targeting range (which could potentially permit single strain targeting)^4^ and the low likelihood of mammalian toxicity.^5^ In addition to the potential value of colicins as bactericidal agents, appropriately engineered colicins have also been demonstrated to have applications in the labelling of *E. coli* cell surfaces. Such colicins are generally mutated to contain a disulfide bond within the receptor binding domain, enabling the colicin to still bind its target receptor at the bacterial cell surface but rendering it incapable of translocating across the bacterial cell envelope and therefore non-bactericidal. Colicins target specific, high abundance receptors on *E. coli* cell surfaces and bind with high affinity, therefore appropriately labelled and engineered colicins are very effective and selective outer membrane-specific probes.^7,8^ For example, colicin Ia binds to the outer membrane iron siderophore transporter Cir with a reported *K*_assoc_ of 1 × 10^10^ M^−1^ at 37 °C,^9^ and there are approximately 5000 copies of the Cir receptor in the outer membrane of each *E. coli* bacterium.^9^ These characteristics enabled colicin Ia labelled with a fluorescent dye to be used as a probe to decipher fundamental mechanisms of outer membrane protein turnover.^7^ Additionally, we have recently demonstrated that small molecules can be expediently appended to colicin E9 using aldehyde based bioconjugation which afford bioconjugates that induce *E. coli* aggregation, through dual targeting of adjacent *E. coli* cell surfaces.^10^ Herein, we present an extension of this approach with colicin Ia, utilising organocatalyst-mediated protein aldol ligation (OPAL)^11^ to target the N-terminus, or maleimide ligation to target an internal cysteine for bioconjugation, culminating in the preparation of mannose-presenting colicin la neoglycoproteins that could have applications in non-genetic glyco-engineering of bacterial cell surfaces.

## 2. Experimental section

Please see ESI† for general methods and materials, LCMS methods, uncropped SDS PAGE gels, lectin blots, western blots and related protocols.

### Synthesis of a biotin-linked colicin Ia conjugate 3 (Scheme S1†)

β-Mercaptoethanol (stock 137 mM in water, 10 μL, final concentration 10% (v/v)) was added to a solution of colicin Ia (100 μL of 32 μM stock in 25 mM phosphate buffer (PB) pH 7.5) in a 0.5 mL micro-centrifuge tube, the solution was mixed by pipette tip swirling and left at RT for 5 min. l-Methionine (1 μL of 22 mM stock in 0.1 M PB, 0.1 NaCl, pH 7.0) and NaIO_4_ (1 μL of 11 mM stock in 0.1 M PB, 0.1 NaCl, pH 7.0) were added to the reaction mixture. The solution was mixed by gentle pipette tip swirling and allowed to sit on ice in the dark for 4 min. The reaction mixture was immediately purified using a PD SpinTrap G25 desalting column (GE Healthcare Life Sciences), eluting into 100 μL of 25 mM PB pH 7.5. The reaction was charged with (*S*)-(−)-5-(2-pyrrolidinyl)-*H*-tetrazole (2.4 μL of 200 mM stock in 25 mM PB pH 7.5) and biotin OPAL probe^12,13^ (4.8 μL of 4 mM stock in 25 mM PB pH 7.5). The solution was mixed *via* pipette tip swirling and incubated for 1 h at 37 °C. The reaction mixture was purified using a PD SpinTrap G25 desalting column (GE Healthcare Life Sciences), eluting into 20 mM potassium phosphate, 500 mM NaCl pH 7.0 for analysis and further manipulation.

### Synthesis of protected mannose-(Gly-Ser)_6_-linked OPAL probe S2

H-Gly-2-ClTrt resin was weighed out into an SPPS cartridge fitted with a PTFE stopcock, swollen in DMF for 30 min and then filtered.

DIPEA (98 μL, 0.56 mmol, 11 eq.) was added to a solution of Fmoc-propargyl-Gly-OH (89 mg, 0.26 mmol, 5 eq.) and HTCU (107 mg, 0.26 mmol, 5 eq.) dissolved in the minimum volume of DMF. The resultant solution was then immediately added to the resin. The reaction mixture was gently agitated by rotation for 1 h and the resin was filtered off and washed with DMF (3 × 2 min with rotation). A solution of 20% (v/v) piperidine in DMF was added to the resin and gently agitated by rotation for 2 min. The resin was filtered off and this process was repeated four more times, followed by washing with DMF (5 × 2 min with rotation).

(i) DIPEA (98 μL, 0.56 mmol, 11 eq.) was added to a solution of Fmoc-Gly-OH (79 mg, 0.26 mmol, 5 eq.) and HTCU (107 mg, 0.26 mmol, 5 eq.) dissolved in the minimum volume of DMF. The resultant solution was then immediately added to the resin. The reaction mixture was gently agitated by rotation for 1 h and the resin was filtered off and washed with DMF (3 × 2 min with rotation). A solution of 20% (v/v) piperidine in DMF was added to the resin and gently agitated by rotation for 2 min. The resin was filtered off and this process was repeated four more times, followed by washing with DMF (5 × 2 min with rotation).

(ii) DIPEA (98 μL, 0.56 mmol, 11 eq.) was added to a solution of Fmoc-Ser(*t*Bu)-OH (101 mg, 0.26 mmol, 5 eq.) and HTCU (107 mg, 0.26 mmol, 5 eq.) dissolved in the minimum volume of DMF. The resultant solution was then immediately added to the resin. The reaction mixture was gently agitated by rotation for 1 h and the resin was filtered off and washed with DMF (3 × 2 min with rotation). A solution of 20% (v/v) piperidine in DMF was added to the resin and gently agitated by rotation for 2 min. The resin was filtered off and this process was repeated four more times, followed by washing with DMF (5 × 2 min with rotation). Steps (i) and (ii) were repeated a further five times.

DIPEA (98 μL, 0.56 mmol, 11 eq.) was added to a solution of (*S*)-2-(4-((3-(*tert*-butoxycarbonyl)-2,2-dimethyloxazolidin-4-yl)methyl)phenoxy)acetic acid^12^ (102 mg, 0.26 mmol, 5 eq.) and HTCU (107 mg, 0.26 mmol, 5 eq.) dissolved in the minimum volume of DMF. The resultant solution was then immediately added to the resin. The reaction mixture was gently agitated by rotation for 1 h and the resin was filtered off and washed with DMF (5 × 2 min with rotation).

DIPEA (1 mL, 5.75 mmol, 55 eq.) was added to a solution of mannose azide 4 (46 mg, 0.105 mmol, 1 eq.), sodium absorbate (31 mg, 0.156 mmol, 1.5 eq.) and copper iodide (60 mg, 0.315 mmol, 3 eq.) dissolved in DMF (2.5 mL), and the solution was added to resin. The reaction mixture was gently agitated by rotation for 12 h and the resin was filtered off and washed sequentially with H_2_O (3 × 2 min with rotation), iPrOH (3 × 2 min with rotation), DMF (3 × 2 min with rotation), iPrOH (3 × 2 min with rotation), and DMF (3 × 2 min with rotation). The resin was washed with DCM (3 × 2 min with rotation) and MeOH (3 × 2 min with rotation). The resin was dried on a vacuum manifold and further dried on a high vacuum line overnight. A solution of cleavage cocktail (95 : 2.5 : 2.5 TFA : H_2_O : triisopropylsilane) was added to the resin and gently agitated by rotation for 1 h. The reaction mixture was drained into ice-cold Et_2_O and centrifuged at 4000 rpm at 4 °C until pelleted (*ca*. 5–10 min). The supernatant was carefully decanted and then subsequently resuspended, centrifuged and supernatant decanted three more times. The precipitated peptide pellet was dissolved in water and lyophilised to obtain a powder. Probe S2 was purified using Bio-Gel P-2 resin (BioRad) in water.

### Synthesis of aldehyde mannose-(Gly-Ser)_6_-linked OPAL probe 7

To a solution of mannose-based inhibitor linked (Gly-Ser)_6_ OPAL probe S2 (160 μL, 15 mM, 4 mg, in 0.1 M PB, 0.1 M NaCl, pH = 7.0) was added NaIO_4_ (22 μL, 112 mM, in 0.1 M PB, 0.1 M NaCl, pH = 7.0) in two 11 μL increments. The reaction was mixed thoroughly and allowed to sit for 3 min on ice in the dark. The solution was then loaded onto a solid-phase extraction cartridge (Grace Davison Extract Clean, 8 mL reservoir, Fisher Scientific) equilibrated with water/acetonitrile. After initial washing with water, the product was eluted over a gradient of acetonitrile. The product was then diluted with water, and subsequently lyophilised to afford 7 as a pale yellow, fluffy powder which was used without further purification (3.8 mg, 96%).

### Synthesis of mannose-(Gly-Ser)_1/3/6_ linked colicin Ia conjugates 8/9/10 (Scheme S2†)

β-Mercaptoethanol (137 mM stock in water, 10 μL, final concentration 10% (v/v)) was added to a solution of colicin Ia (100 μL of 100 μM stock in 25 mM PB pH 7.5) in a 0.5 mL micro-centrifuge tube, mixed by pipette tip swirling and left at RT for 5 min. The solution was charged with l-methionine (1 μL of 66 mM stock in 0.1 M PB, 0.1 NaCl, pH 7.0) and NaIO_4_ (1 μL of 33 mM stock in 0.1 M PB, 0.1 NaCl, pH 7.0) and mixed by gentle pipette tip swirling. The solution was incubated on ice in the dark for 4 min. The reaction mixture was immediately purified using a PD SpinTrap G25 desalting column (GE Healthcare Life Sciences), eluting into 100 μL of 25 mM PB pH 7.5. The reaction mixture was charged with (*S*)-(−)-5-(2-pyrrolidinyl)-*H*-tetrazole (20 μL of 200 mM stock in 25 mM PB pH 7.5) and probe 5 or 6^10^ (40 μL of 4 mM stock in 25 mM PB pH 7.5) or 7 (27 μL of 4 mM stock in 25 mM PB pH 7.5). The solution was mixed *via* pipette tip swirling and incubated for 1 h at 37 °C. The reaction mixture was purified using a PD SpinTrap G25 desalting column (GE Healthcare Life Sciences), eluting into 20 mM potassium phosphate, 500 mM NaCl pH 7.0.

### Synthesis of *N*-Boc-1,2-diaminoethane 11 (ref. 14)

To a solution of 1,2-diaminoethane (3 mL, 0.045 mol, 10 eq.) in DCM (100 mL) was added dropwise at 0 °C a solution of di-tertbutyldicarbonate (1 g, 4.5 mmol) in DCM. The reaction mixture was stirred at RT for 16 h, affording a cloudy white solution. The solution was washed with brine (6 × 100 mL) and water (1 × 100 mL), dried (MgSO_4_) and concentrated *in vacuo* to yield a colourless oil of *N*-Boc-1,2-diaminoethane 11 (0.59, 3.7 mmol, 81%).

^1^H NMR (500 MHz, CDCl_3_) *δ* 5.13 (s, 1H, NH), 3.15 (m, 2H, CH_2_), 2.77 (t, *J* = 5.9 Hz, 2H, CH_2_), 2.18 (s, 2H, NH_2_), 1.41 (s, 9H, CH_3_).

^13^C NMR (126 MHz, CDCl_3_) *δ* 156.3 (1C, C=O), 79.3 (1C, C), 43.1 (1C, CH_2_), 41.8 (1C, CH_2_), 28.5 (s, 3C, CH_3_).

(ESI)HRMS – C_7_H_16_O_2_NaN_2_^+^ ([M + Na]^+^) requires *m*/*z* 183.2068: found *m*/*z* 183.1104.

IR – 3354 (N–H), 2975 (C–H), 2932 (C–H), 2864 (C–H), 1681 (C=O), 1161 (C–O).

### Synthesis of Boc-protected mannosyl amine S1 (Scheme S3†)

*N*-Boc-1,2-diaminoethane 11 (0.21 g, 1.3 mmol, 2 eq.) was added to a solution of 12 ref (0.25 g, 0.65 mmol) and NEt_3_ (0.380 mL) in dry methanol (2 mL). The reaction mixture was stirred at RT for 16 h forming a cloudy white solution. The solution was neutralized with Dowex, filtered and concentrated *in vacuo*, affording a white solid. The solid was purified using column chromatography, eluting with a solvent system of 3 : 1 EtOAc : MeOH. This afforded a colourless solid of S1 (0.13 g, 0.28 mmol, 43%); *R*_f_ = 0.31 (2 : 1 hexane : EtOAc).

^1^H NMR (500 MHz, DMSO) *δ* 10.01 (s, 1H, NH), 7.95 (s, 1H, NH), 7.37 (d, *J*_HAr_ = 9.0 Hz, 2H, HAr), 7.05 (d, *J*_HAr_ = 9.0 Hz, 2H, HAr), 6.94 (s, 1H, NH), 5.28 (d, *J*_1,2_ = 1.6 Hz, H-1), 4.98 (d, *J*_2,OH_ = 4.0 Hz, 1H, OH (H-2)), 4.81 (d, *J*_4,OH_ = 5.6 Hz, 1H, OH (H-4)), 4.72 (d, *J*_3,OH_ = 5.3 Hz, 1H, OH (H-3)), 4.45 (t, *J*_6,OH_ = 5.7 Hz, 1H, OH (H-6)), 3.81 (s, 1H, H-2), 3.66 (m, 1H, H-3), 3.63–3.55 (m, 3H, CH_2,_ H-6a), 3.50–3.40 (m, 3H, H-4, H-5, H-6b), 3.14 (dd, *J*_CH2_ = 11.9 Hz, 6.2 Hz, 2H, CH_2_), 1.35 (s, 9H, CH_3_).

^13^C NMR (126 MHz, DMSO) *δ* 183.3 (1C, C=O), 180.4 (1C, C=O), 169.4 (1C, C=O), 164.7 (1C, C), 155.8 (CAr), 152.2 (1C, C), 133.7, 119.1, 117.9 (CAr), 99.5 (1C, C-1), 77.8 (1C, C), 74.9 (1C, C-5), 70.9 (1C, C-3), 70.1 (1C, C-2), 66.8 (1C, C-4), 61.1 (1C, C-6), 43.7 (1C, CH_2_), 41.0 (1C, CH_2_), 28.2 (3C, CH_3_).

(ESI)HRMS – C_23_H_31_N_3_NaO_10_^+^ ([M + Na]^+^) requires *m*/*z* 532.1907: found *m*/*z* 532.1916[*α*]^25^_D_ = +73.22 (*c* 1, DMSO)

IR – 3292 (O–H), 3210 (N–H), 2977 (C–H), 2933 (C–H), 1795 (C=O), 1671 (C=O), 1577 (C=C), 1231 (C–O),1004 (C–O).

### Synthesis of mannosyl amine 13 (ref. 15)

S1 (0.08 g, 0.16 mmol), was dissolved in a 1 : 1 solution of H_2_O : TFA (4 mL : 4 mL) affording a clear solution which was stirred at RT for 3 h. The solution was concentrated *in vacuo*, azeotroping with toluene to yield a clear gel. The gel was dissolved in MeOH and neutralised with a free base resin. The resin was removed *via* filtration to leave a clear solution which was concentrated *in vacuo* to yield a crude colourless solid containing 13 (52 mg, ≤0.13 mmol, ≤81%).

^1^H NMR (500 MHz, DMSO) *δ* 7.37 (d, *J*_HAr_ = 8.8 Hz, 2H, HAr), 7.06 (d, *J*_HAr_ = 8.8 Hz, 2H, HAr), 5.29 (d, *J*_1,2_ = 1.5 Hz, 1H, H-1), 3.82 (dd, *J*_2,3_ = 3.0 Hz, *J*_1,2_ = 1.5 Hz, 1H, H-2), 3.67 (m, 3H, CH_2_, H-3), 3.60 (dd, *J* = 11.5 Hz, *J* = 1.5 Hz, 1H, H-6a), 3.52–3.45 (m, 2H, H-6b, H-4), 3.42 (m, 1H, H-5), 2.88 (t, *J*_CH2_ = 5.9 Hz, 2H, CH_2_).

^13^C NMR (126 MHz, DMSO) *δ* 183.7 (1C, C=O), 180.6 (1C, C=O), 169.4 (1C, C), 164.4 (1C, C), 152.4, 133.9, 119.6, 118.0 (CAr), 99.6 (1C, C-1), 74.9 (1C, C-5), 70.8 (1C, C-3), 70.2 (1C, C-2), 66.9 (1C, C-4), 61.2 (1C, C-6), 44.3 (1C, CH_2_), 41.2 (1C, CH_2_).

(ESI)HRMS – C_18_H_23_N_3_NaO_8_^+^ ([M + Na]^+^) requires *m*/*z* 432.1383: found *m*/*z* 432.1377.

IR – 3363 (O–H), 3249 (O–H), 2946 (C–H), 2907 (C–H), 1799 (C=O), 1652 (C=O), 1546 (C=C), 1511 (C=C), 1436 (C=C), 1183 (C–O), 1124 (C–O).

### Synthesis of a mannose-linked colicin Ia conjugate 16 *via* maleimide ligation (Scheme S4†)

Mannosyl amine 13 (stock 1 M in DMSO, 3 μL, final concentration 500 mM) was added to bifunctional NHS-(PEG)_8_-maleimide linker 14 (stock 500 mM in DMSO, 3 μL, final concentration 250 mM) in a 0.5 mL micro-centrifuge tube. The solution was mixed by swirling the pipette tip and was incubated in the dark at RT for 30 min. The reaction mixture was added to a solution of colicin Ia (100 μL, 100 μM in 1 × PBS pH 7.2) which had been pre-treated with β-mercaptoethanol *via* 3 × 2 μL additions of β-mercaptoethanol stock over 30 min (stock 137 mM in water, final concentration 10% (v/v)), while being incubated in the dark at RT, the reaction was purified using a PD SpinTrap G25 desalting column (GE Healthcare Life Sciences), eluting into 20 mM potassium phosphate, 500 mM NaCl pH 7.0.

## 3. Results and discussion

**Fig. 1:**
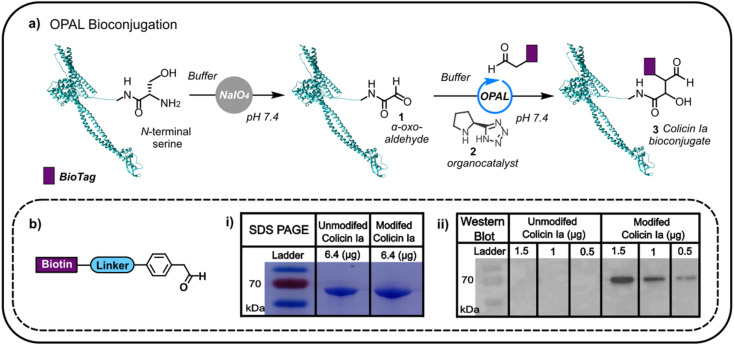
(a) The oxidation of the N-terminal serine residue of colicin Ia (PDB; 1CII) can be used to install an α-oxo aldehyde motif. This motif can be site-selectively targeted using proline tetrazole catalysed OPAL to append a biologically relevant tag (BioTag) to the N-terminus of colicin Ia. (b) A depiction of the biotin-linked OPAL probe, (i) SDS-PAGE analysis of biotin-labelled colicin Ia and (ii) western blot analysis of biotin-labelled colicin Ia.

Colicin Ia has three distinct domains: an N-terminal translocation domain, a receptor binding domain and a C-terminal channel forming domain.^16^ As the N-terminus is distant from the domain through which colicin la binds the Cir receptor, we initially focused on N-terminal modification anticipating that distal functionalisation would be less likely to disrupt binding to Cir receptor at the cell surface. This N-terminal modification would enable us to append glycans and other biomolecules to the protein, which would then anchor to the outer membrane, potentially facilitating non-genetic reengineering of bacteria by colicin Ia-mediated cell surface presentation. Colicin la has an N-terminal serine residue which we recognised could be site-specifically oxidised with NaIO_4_ to conveniently introduce a bioorthogonal α-oxo aldehyde, amenable to subsequent modification using aldehyde selective bioconjugation. Colicin Ia was therefore treated with NaIO_4_, and the successful introduction of an α-oxo aldehyde 1 verified by functionalisation with a biotin-functionalised probe^12,13^ (Fig. 1a) *via* OPAL, a bioconjugation that uses a proline tetrazole organocatalyst 2 to accelerate a cross aldol reaction between a small molecule aldehyde and a protein α-oxo aldehyde.^11^ Colicin Ia has a large molecular weight of ∼70 kDa,^17^ which makes it extremely challenging to analyse by protein mass spectrometry, even using Fourier-transform based methods^18^ and top-down proteomics,^19^ and although the small mass increase resulting from the coupling of the biotin-functionalised probe (approximately 1 kDa) was also difficult to observe in a Coomassie-stained SDS-PAGE gel as a shift in band migration (Fig. 1b(i)), western blotting provided evidence that the biotin moiety had been successfully ligated to the OPAL modified protein 3 (Fig. 1b(ii)).

**Fig. 2:**
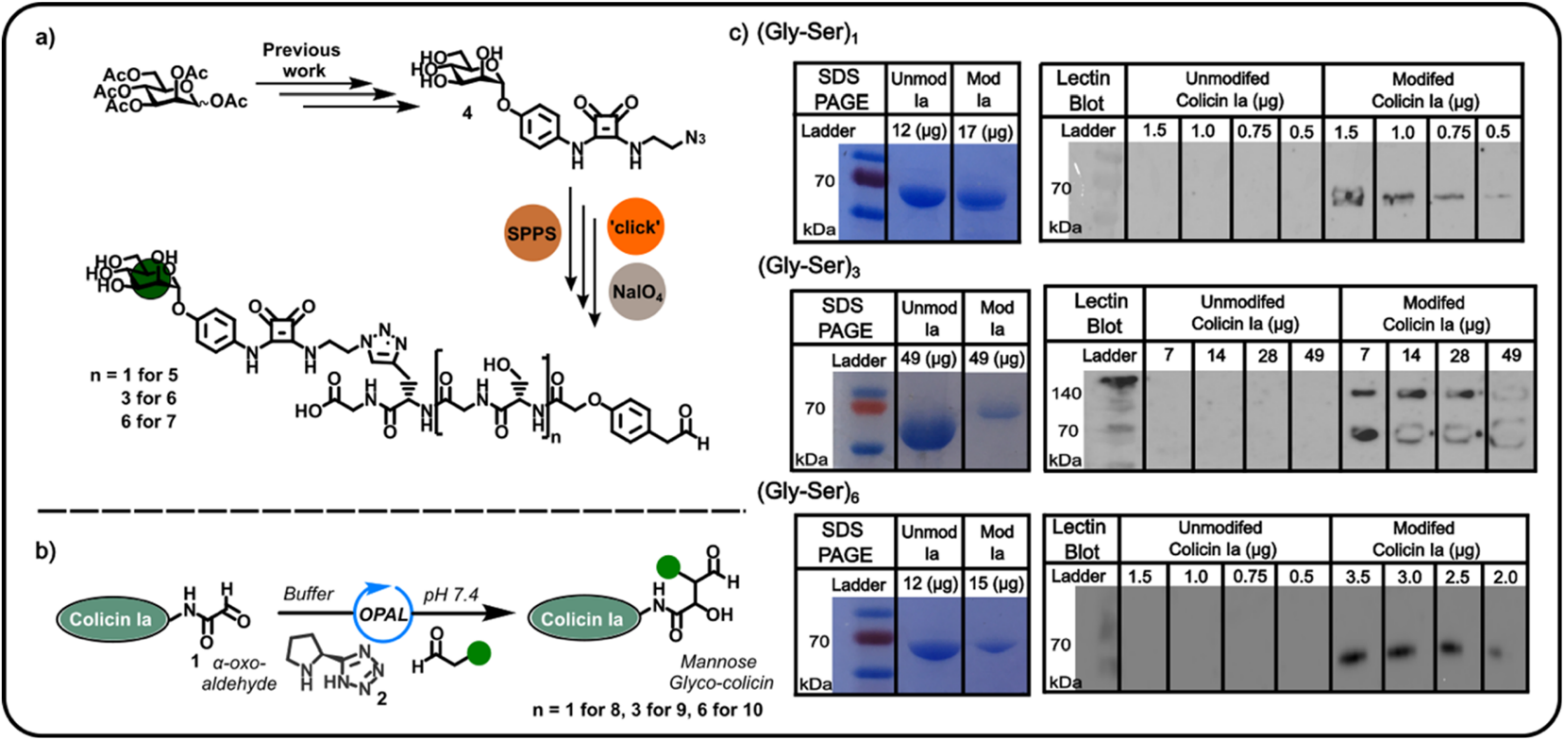
(a) Incorporation of the previously reported mannose azide 4 into mannose containing OPAL probes 5–7. (b) A schematic illustration of the OPAL bioconjugation of an α-oxo colicin Ia 1 with mannose-bearing OPAL probes 5–7. (c) SDS PAGE and concanavalin A lectin blot analyses of the mannose-functionalised colicin Ia conjugates (Gly-Ser)_1_8, (Gly-Ser)_3_9 and (Gly-Ser)_6_10 demonstrating successful bioconjugation.

Confident in the bioconjugation approach, we then used an azide-functionalised α-mannoside 4 (Fig. 2) to construct α-mannose functionalised OPAL probes previously shown to bind to FimH, an *E. coli* surface lectin which mediates the binding of pathogenic adherent bacteria to human cells.^20^ We anticipated these probes, and likewise other glycans, could be used in the construction of colicin neoglycoproteins with utility in glyco-engineering of bacterial cell surfaces. As the length of the linker between the colicin and the glycan could be expected to influence the biological properties of the resultant neoglycoprotein, we used repeating units of serine and glycine as a linker, facilitating easy tailoring of OPAL probe length. A total of three α-mannose-presenting OPAL probes 5–7 of varying linker length were prepared using a combination of solid-phase peptide synthesis (SPPS), click chemistry, and NaIO_4_ oxidation,^10^ including probe 7 with an extended (Gly-Ser)_6_ linker. Each probe was then used in the bioconjugation of α-oxo aldehyde colicin la 1 under the established OPAL conditions. The resultant α-mannose colicin la neoglycoproteins 8–10 were subsequently analysed using SDS-PAGE and lectin blot (Fig. 2), employing concanavalin A (a lectin which binds terminal α-mannose residues).^21^ While SDS-PAGE analysis of all three colicin la neoglycoproteins showed little discernible decrease in band migration compared to unmodified colicin Ia, which was likely due to the relatively small mass addition of each probe (approximately 1–1.6 kDa). Lectin blot analysis clearly indicated that all the α-mannose presenting OPAL probes, 5–7, were successfully conjugated to colicin la, with no immunoblotting activity discernible in lanes containing unmodified colicin Ia, and all lanes containing colicin Ia samples subjected to OPAL with α-mannose presenting probes displaying a clear band at approximately 70 kDa (Fig. 2). The site-directed colicin Ia mutant used in our bioconjugation experiments contains a non-native, surface-exposed Cys residue in its C-terminal domain (Lys mutated to Cys at position 544). Evidence that this residue is unperturbed by the bioconjugation and free to form inter-protein disulfide bonds is presented in lectin blot and SDS-PAGE analyses of colicin la modified with the (Gly-Ser)_3_ probe 6 under non-reducing conditions, where a protein dimer band at approximately 140 kDa is observed (Fig. 2).

**Fig. 3:**
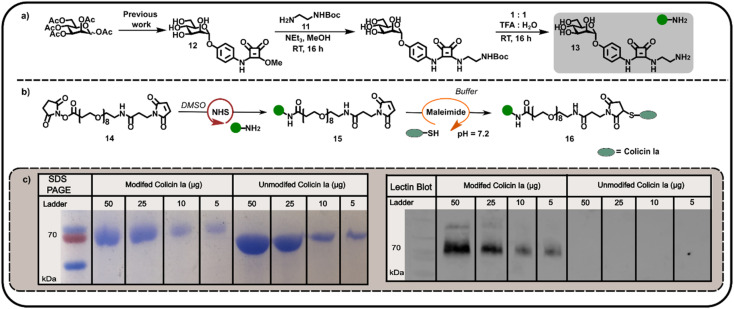
(a) Synthesis of mannose amine 13 from mannose squarate ester precursor 12. Reagents and conditions: (i) *N*-Boc-1,2-diaminoethane, NEt_3_, MeOH, room temperature (RT), 16 h, 43% (ii) 1 : 1 H_2_O : TFA, RT, 3 h, 81%. (b) A schematic depiction of formation of maleimide-functionalised mannoside 15 using NHS-ligation of mannose amine 13 to a heterobifunctional linker 14, and subsequent maleimide ligation to afford mannose-functionalised colicin Ia 16. (c) SDS PAGE and concanavalin A lectin blot analyses of the mannose colicin Ia neoglycoprotein 16 prepared by maleimide ligation.

We subsequently explored the reactivity of this surface-exposed Cys residue in the construction of alternative conjugates, wherein the mannose glycans may be presented in a different orientation to N-terminally modified neoglycoproteins. In order to construct an appropriate Cys-reactive mannose functionalised probe, *N*-Boc-1,2-diaminoethane 11 was reacted with mannose squarate ester 12 (Fig. 3a), followed by Boc deprotection to afford amine functionalised mannoside 13. We then used amine 13 to derivatise heterobifunctional linker (NHS-ester to maleimide) 14 in DMSO (Fig. 3b), affording a maleimide functionalised α-mannoside 15. Maleimide 15 was then added to a solution of colicin Ia in three equal portions over 30 min, at pH 7.2 in order to favour selective Michael addition between the surface-exposed Cys and the maleimide. As with previous bioconjugation experiments, the successful attachment of maleimide functionalised α-mannoside 15 to colicin Ia to afford mannose colicin Ia neoglycoprotein 16 was confirmed using SDS-PAGE and lectin blot analyses (Fig. 3c). A subtle decrease in band migration for mannose-functionalised colicin Ia compared to unmodified colicin Ia was observed, which was reinforced by the successful concanavalin A lectin blot analysis (Fig. 3c). Although it should be noted that in these examples SDS-PAGE and blot analysis is not quantitative.

## 4. Conclusion

In conclusion, we have presented two methods for the preparation of mannose-presenting neoglycoproteins of colicin la. Specifically, the construction of three N-terminal modified mannose conjugates further expands the use of OPAL ligation with latent electrophilic α-oxo aldehydes which can be installed into colicin proteins through selective N-terminal NaIO_4_ oxidation. Furthermore, a heterobifunctional linker containing both an NHS-ester and a maleimide could also be used for the assembly of mannose neoglycoproteins, exploiting the nucleophilic reactivity of an engineered, surface-exposed Cys residue in the C-terminal domain of colicin Ia. Notably, the orthogonal reactivity of these two approaches, which enable colicin Ia modification on the N-terminal translocation and *C*-terminal cytotoxic domains respectively, may be harnessed in future studies to afford dual functionalised bioconjugates able to bind bacterial cell surfaces through their unencumbered receptor binding domain. Ultimately, the mild reaction conditions and flexibility of both bioconjugations make them ideal tools for colicin Ia modification with capricious biomolecules such as glycans. Akin to our previous studies with colicin E9 (ref. 10), in future work we aim to demonstrate that the colicin Ia neoglycoproteins disclosed herein will enable non-genetic glyco-engineering of bacterial cell surfaces.

## Data availability

The data supporting this article have been included as part of the ESI.†

## Author contributions

Natasha Hatton: investigation, methodology, visualisation, writing – original draft, conceptualization. Laurence Wilson: supervision, funding acquisition. Christoph Baumann: supervision, funding acquisition, conceptualization, writing – review & editing. Martin Fascione: supervision, funding acquisition, conceptualization, writing – original draft, writing – review & editing, project administration.

## Conflicts of interest

There are no conflicts to declare.

## Supplementary Material

RA-014-D4RA04774E-s001
