# Supplementary material for: Synthesis of colicin Ia neoglycoproteins: tools towards glyco-engineering of bacterial cell surfaces
Source: RSC Adv. 2024 Sep 13;14(40):29106–12. doi: 10.1039/d4ra04774e (PMC11394469; doi:10.1039/d4ra04774e)
Supplement: RA-014-D4RA04774E-s001 [file RA-014-D4RA04774E-s001.pdf]

Supplementary Information (SI) for RSC Advances.
This journal is © The Royal Society of Chemistry 2024

Synthesis of Colicin Ia Neoglycoproteins: Tools Towards Glyco-Engineering of Bacterial Cell Surfaces

Natasha E. Hatton,[a] Laurence G. Wilson,[b] Christoph G. Baumann*[c] and Martin A. Fascione*[a]

[a] Department of Chemistry, University of York, York, YO10 5DD, UK; [b] School of Engineering, Physics and Technology, University of York, York, YO10 5DD, UK; [c] Department of Biology, University of York, York, YO10 5DD, UK

Email: martin.fascione@york.ac.uk; christoph.baumann@york.ac.uk

Supporting information

General Appendix

Non-chapter specific experimental procedures/information

The use of dry solvents is stated in individual methodologies. All dry solvents used were dried according to standard methods and solvents used for flash chromatography purposes were not dried prior to use. All chemical synthesis reactions were carried out in oven-dried glassware. Thin layer chromatography was carried out on Merck silica gel 60 F_{254} pre-coated aluminium foil sheets and were visualised using UV light (254 nm) and stained with either a 5% (v/v) sulphuric acid in EtOH stain (for sugars), or a Ninhydrin stain (for amines and BOC protected amines).

Spectroscopic and spectrometric instruments and standard practices

^{1}H-NMR (500 MHz) and ^{13}C-NMR (126 MHz) experiments were conducted using a Bruker AVIIIHD 500 instrument at The University of York Centre for Magnetic Resonance. ^{1}H-NMR (400 MHz) and ^{13}C-NMR (101 MHz) experiments were conducted using a JEOL 400 instrument at The University of York Centre for Magnetic Resonance. Multiplicities are given as singlet (s), doublet (d), triplet (t), doublet of doublets (dd), doublet of doublet of doublets (ddd), triplet of doublets (td), quartet of doublets (qd) or multiplet (m). Resonances were assigned using HH-COSY and CH-HSQC. All NMR chemical shifts (δ) were recorded in ppm and coupling constants (J) are reported in Hz. Topspin 4.0.6 and MestReNovax64 were primarily used for processing the spectral data. The notation of the chemical environments to which the ^{1}H and ^{13}C NMR data of monosaccharides are assigned are described in Figure S1.

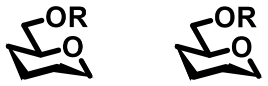

Figure S1. Depicts chemical environment for ^{1}H and ^{13}C assignment

Fourier transform infrared (FTIR) spectra were recorded on a PerkinElmer UATR 2 spectrometer using the attenuated total reflectance (ATR) technique. Optical rotations were measured using a Bellingham and Stanley ADP 450 Automatic Digital Peltier Controlled Polarimeter equipped with a 589 nm LED. Concentration is denoted as “c” and was calculated as grams per 100 millilitres (g / 100 mL) whereas the solvent is indicated in parenthesis (c, solvent).

ESI-MS experiments used for the characterisation of chemically synthesised molecules were conducted using a Bruker micrOTOF mass spectrometer coupled to an Agilent 1200 series LC system. More details regarding the ESI-LC/MS analysis of OPAL probes and enzymatically synthesised sugars can be found below.

LCMS Procedures

ESI-LC/MS of OPAL probes

LC-MS analysis was performed on a Dionex UltiMate® 3000 Ci Rapid Separation LC system equipped with an UltiMate® 3000 photodiode array detector probing at 250-400 nm coupled to a HCT ultra ETD 11 (Bruker Daltonics) ion trap spectrometer, using Chromeleon® 6.80 SR12 software (ThermoScientific), Compass 1.3 for esquire HCT build 581.3, esquire Control version 6.2, Build 62.24 software (Bruker Daltonics) and Bruker compass HyStar 3.2-SR2, HyStar version 3.2, Build 44 software (Bruker Daltonics) at The University of York Centre of Excellence in Mass Spectrometry (CoEMS). Data analysis was performed using ESI compass 1.3 DataAnalysis, Version 4.4 software (Bruker Daltonics).

All peptide/protein mass spectrometry was conducted in positive ion mode unless otherwise stated. Samples made for LCMS were made using a 50% (v/v) HPLC grade water/acetonitrile, 1%formic acid solution.

General analysis of protected and active OPAL probes

Samples were analysed using an Accucore™ C18 HPLC Columns 2.6 µm 2.1 x 150 mm reverse-phase column. Water + 0.1% formic acid by volume (solvent A) and acetonitrile + 0.1% formic acid (solvent B) were used as a mobile phase at a flow rate of 300 µL min^{-1} at room temperature (RT). A multi-step gradient of 7.5 min was programmed as follows: 95% A for 1.0 min, followed by a linear gradient to 95% B over 6.5 min, followed by 95% B for an additional 1.0 min. A linear gradient to 95% A was used to equilibrate the column.

SDS-PAGE gel and lectin blotting protocols

SDS-PAGE gel protocol

10% (w/v) SDS-PAGE gels were used to analyse bioconjugation. These 10% (w/v) SDS-PAGE gels were poured in-house using a specialised kit, and were made using the following protocol: H_2O (4 mL) was mixed with 2.5 mL of resolving buffer (1.5 M Tris-HCl, 0.4% (w/v) SDS, pH 8.8). To the resultant mixture was added 30% (w/v) acrylamide:0.8% (w/v) bis-acrylamide (37.5:1) (3.3 mL), 20% (w/v) ammonium persulfate solution (50 µL) and tetramethylethylenediamine (10 µL). The resultant mixture was briefly gently agitated to ensure mixing, and then poured into the mould and allowed to set. Once the gel had set a stacking gel was allowed to set on top of the main gel, with a comb inserted to create sample lanes. The protocol for mixing the stacking gel is as follows: H_2O (3.2 mL) was mixed with 1.3 mL of resolving buffer. To the resultant mixture was added 30% (w/v) acrylamide:0.8% (w/v) bis-acrylamide (37.5:1) (0.5 mL), 20% (w/v) ammonium persulfate solution (12.5 µL) and tetramethylethylenediamine (8 µL). Once the SDS-PAGE gels were fully prepared, complete with stacker gel and sample lanes, the SDS-PAGE gels were ready to be loaded with samples. Unless otherwise stated, samples were mixed with a 5 × concentrated reducing buffer (10% (w/v) SDS, 10 mM 2-mercaptoethanol, 20% (w/v) glycerol, 200 mM Tris-HCl pH 6.8, 0.05% (w/v) bromophenol blue) and boiled for 5 min prior to running on the SDS-PAGE gel. The molecular weight markers used were PageRuler™ Plus Prestained Protein Ladder (ThermoFisher Scientific). Each gel was run at 200 V for 30-80 min in SDS running buffer (25 mM Tris, 192 mM glycine, not pH adjusted). After being run, SDS-PAGE gels were fixed via gel immersion in a fixing solution (40% water, 50% EtOH and 10% AcOH by volume) which was gently rocked for 60 min. For experiments in which the SDS PAGE gel was subsequently stained with Coomassie stain, the fixed gels were then immersed in a solution of 0.1% (w/v) Coomassie Brilliant Blue R-250 (in 40% water, 50% EtOH and 10% AcOH by volume), and the solution brought to the boil in a microwave, before being gently rocked at RT for a further 20 min. The Coomassie stained gels would then be destained via immersion in a destaining solution (50% water, 40% EtOH and 10% AcOH by volume), which was periodically replaced with dye-free destaining solution as the destaining process progressed.

Lectin blot protocol

The blots were assembled using 12 layers of blotting paper and one layer of nitrocellulose membrane which has previously been soaked in transfer buffer (25 mM Tris-HCl pH = 8.3, 192 mM glycine, 20% (v/v) MeOH) for 10 min and an SDS-PAGE gel which had been run but not stained. The blots were assembled in the following order - 6 layers blotting paper, nitrocellulose membrane, 10% (w/v) SDS-

PAGE gel and 6 layers of blotting paper. The gels were transferred to the nitrocellulose membranes using a Trans-Blot®Turbo™ transfer system running at 1.3 A constant; up to 25 V for 30 min. The membranes were then incubated in 1 x PBS and 2% Tween® 20 for 5 min with gentle rocking followed by two further washes with 1 x PBS (5 min with gentle rocking). The membranes were then incubated with 10 mL 1 x PBS containing 0.05% Tween® 20 (Sigma Aldrich), 1 mM $CaCl_2$, 1 mM $MnCl_2$ and 1 mM $MgCl_2$ and 5 pM lectin peroxidase (Sigma Aldrich). The blots were incubated at RT for 16 h and then incubated with 4 mL Amersham (Cytiva) for 5 min. Finally the blots were imaged using a Syngene G:BOX Chemi XRQ equipped with a Synoptic 4.0 MP camera, with GeneSyn software (Version 1.5.70).

Western blot protocol

The blots were assembled using 12 layers of blotting paper and one 10% (w/v) SDS PAGE gel all previously soaked in transfer buffer (25 mM Tris-HCl pH = 8.3, 192 mM glycine, 20% (v/v) MeOH) for 10 min. A further layer of Polyvinylidene difluoride (PVDF) membrane was used which had been soaked in methanol (30 sec), water (30 sec) and transfer buffer (25 mM Tris-HCl pH = 8.3, 192 mM glycine, 20% (v/v) MeOH) (10 min). The blots were assembled in the following order - 6 layers blotting paper, PVDF membrane, 10% (w/v) SDS PAGE gel and 6 layers of blotting paper. The gels were transferred to the PVDF membrane using a Trans-Blot®Turbo™ transfer system running at 1.3 A constant; up to 25 V for 30 min. The membranes were transferred to a 1 x PBS solution 5% (w/v) milk solution and incubated overnight at 4 °C with gentle agitation. The membranes were incubated with either 2.5 µL (1 : 4000) of HRP-Conjugated Streptavidin, (1.25 mg/mL, Thermo Scientific Pierce) or 500 ng of Biotin antibody (HRP) (GeneTex) for 1 h. The membranes were washed twice with 1 x PBS 0.1% Tween® 20 (Sigma Aldrich) and once with 1 x PBS, all washes were performed for 5 min with gentle agitation. The blots were incubated with 4 mL of western blotting detection reagent (Cytiva) for 5 min and the blots were imaged using a Syngene G:BOX Chemi XRQ equipped with a Synoptic 4.0 MP camera, with GeneSyn software (Version 1.5.70).

Colicin Ia sequence, production and purification

The colicin Ia construct used in this work was first published by Kleanthous and co-workers.[1]

Protein Sequence

MSDPVRITNPGAESLGYDSDGHEIMAVDIYVNPPRVDVFHGTPPAWSSFGNKTIWGGNEWVDDSPTRSDIEKRD
KEIAAYKNTLSAQQKENENKRTEAGKRLSAAIAAREKDENTLKTLRAGNADAADITRQEFRLLQAELREYGFRTEIAG
YDALRLHTESRMLFADADSLRISPREARSLIEQAEKRQKDAQNADKKAADMLAEYERRKGILDTRLSELEKNGGAAL
AVLDAQQVRLLGQQTRNDRAISEARNKCSSVTESLNTARNALTRAEQQLTQQKNTPDGKTIVSPEKFPGRSSTNHS
IVVSGDPRFAGTIKITTSAVIDNRANLNYLLTHSGLDYKRNILNDRNPVVTEDVEGDKKIYNAEVAEWDKLRQRLLD
ARNKITSAESAVNSARNNLSARTNEQKHCNDALNALLKEKENIRNQLAGINQKIAEEKRKQDELKATKDAINFTTEF
LKSVSEKYGAKAEQLAREMAGQAKGKKIRNVEEALKTYEKYRADINKKINAKDRAAIAAALESVKLSDISSNLNRFSR
GLGYAGCFTSLADWITEFGKAVRTENWRPLFVKTEAIIAGNAATALVALVFSILTGSALGIIGYGLLMAVTGALIDESL
VEKANKFWGILEHHHHHH

Protein information

Extinction coefficient at 280 nm (calc): 59,360 M^{-1} cm^{-1}

Abs 0.1% (= 1 g/l) 0.842, assuming all Cys residues are reduced

Protein production and purification

The colicin Ia protein was produced recombinantly and purified using a previously published method.[1]

Construction of biotin-linked colicin Ia conjugate **3**

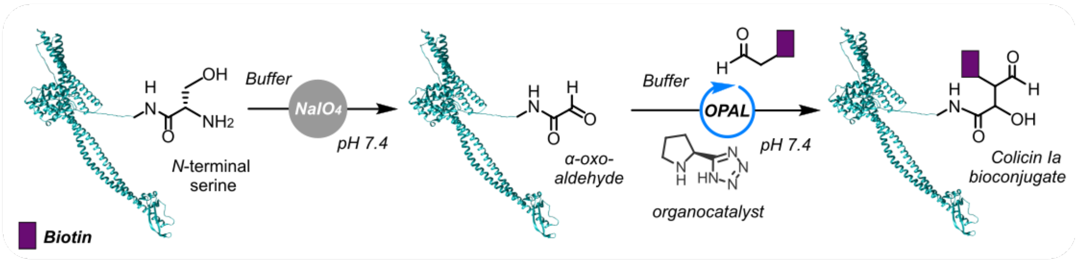

Scheme S1. Depiction of initial $NaIO_4$ oxidation of colicin Ia (PDB; 1CII[2]) followed by OPAL ligation with a biotin probe

Uncropped SDS-PAGE gel and Western blot

SDS-PAGE

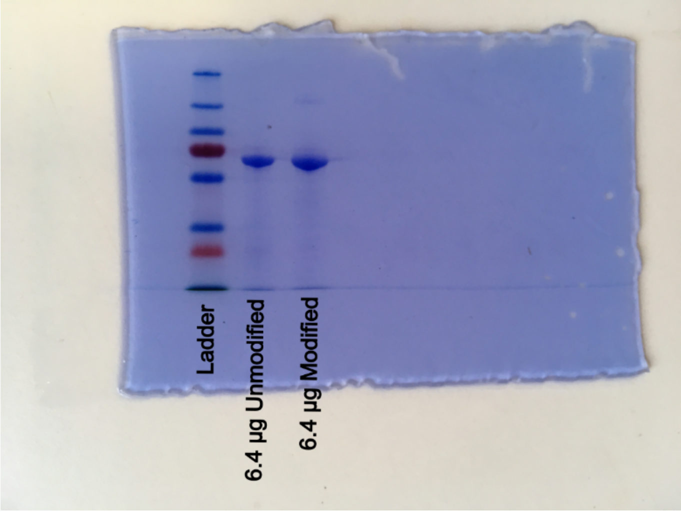

Figure S2. Uncropped SDS PAGE gel of the biotin linked colicin Ia conjugate **3**

Western blot

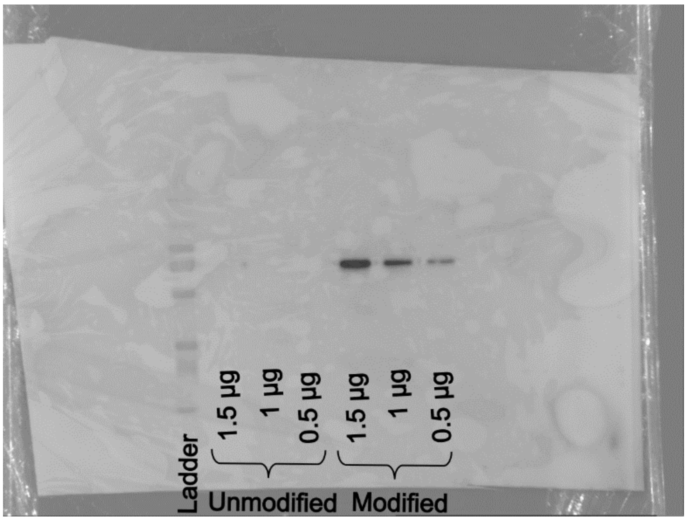

Figure S3. Uncropped Lectin blot of the biotin linked colicin Ia conjugate **3**

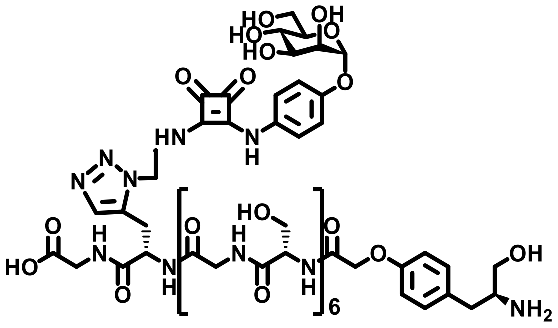

[M+H]$^{+}$: 1678.45

[M+2H]$^{2+}$: 839.57

Figure S4. Structure of protected mannose OPAL probe **S2**

HPLC-MS of protected mannose-(Gly-Ser)$_6$-linked OPAL probe **S2**

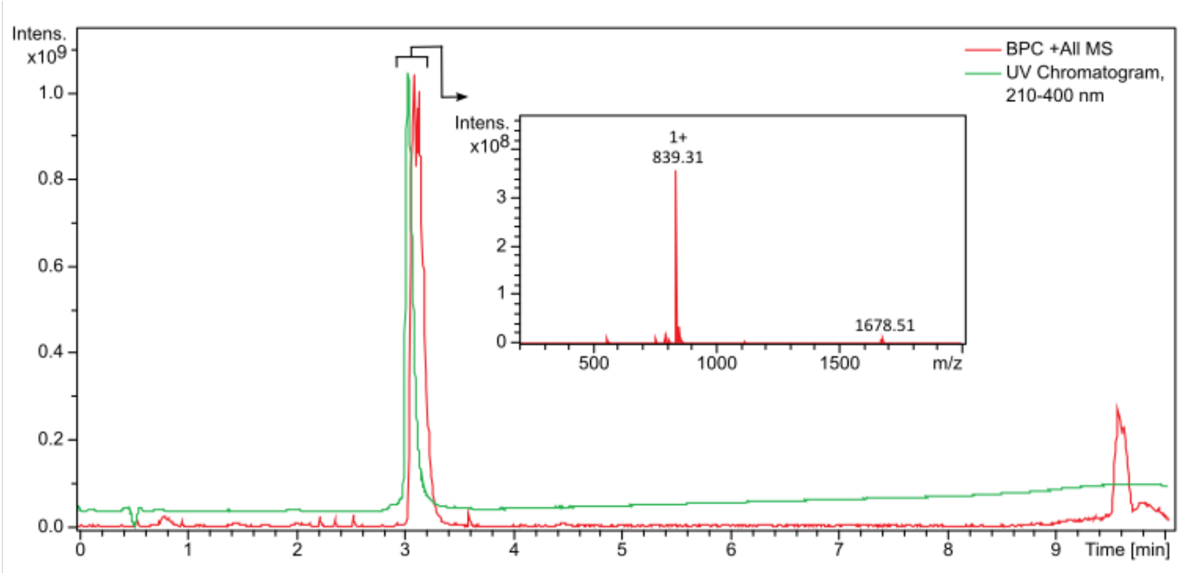

Figure S5. LC trace and mass spectrum of protected mannose-(Gly-Ser)$_6$-linked OPAL probe **S2**

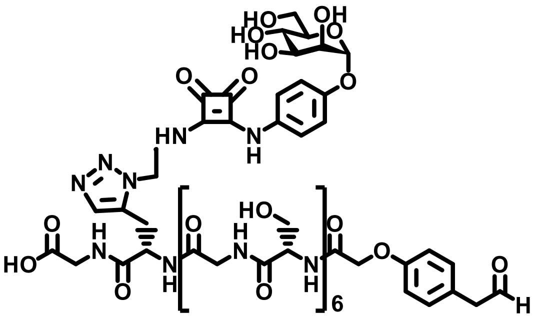

Figure S6. Structure of mannose probe **7**

Synthesis of a mannose-(Gly-Ser)$_1$-linked colicin Ia conjugate 8/9/10

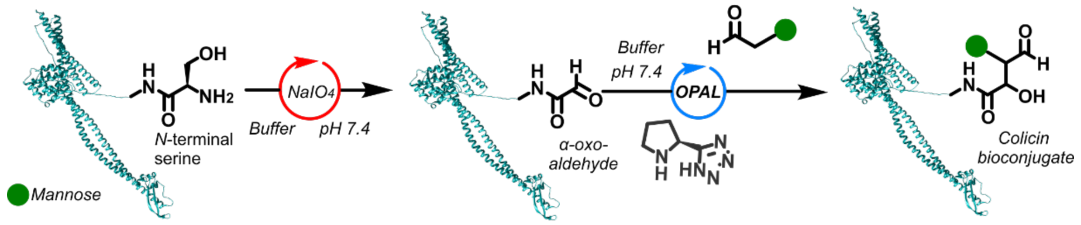

Scheme S2. Depiction of initial $NaIO_4$ oxidation of colicin Ia (PDB; 1CII[2]) followed by OPAL ligation to a mannose linked OPAL probes **5**/**6**/**7**

Uncropped SDS-PAGE gel and Lectin blot

SDS-PAGE gel

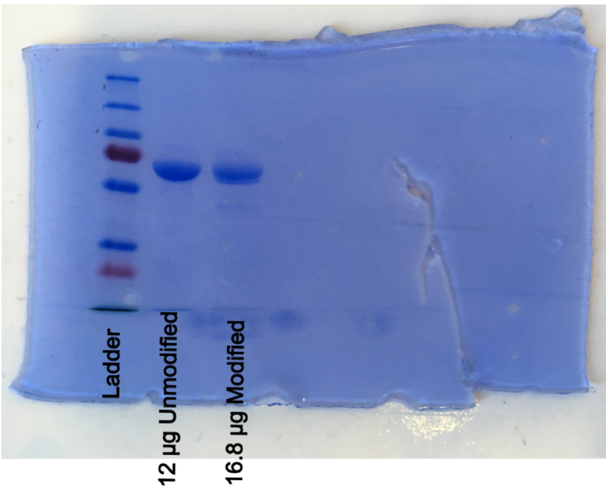

Figure S7. Uncropped SDS PAGE analysis of mannose-(Gly-Ser)-linked colicin Ia conjugate **8**

Lectin Blot

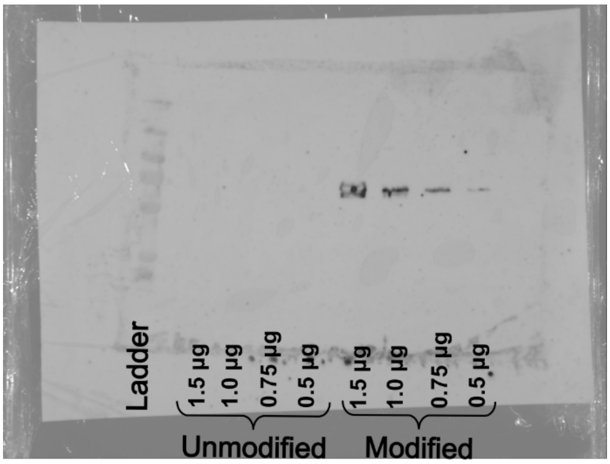

Figure S8. Uncropped Lectin blot analysis of mannose-(Gly-Ser)-linked colicin Ia conjugate **8**

Uncropped SDS-PAGE gel and Lectin blot

SDS-PAGE gel

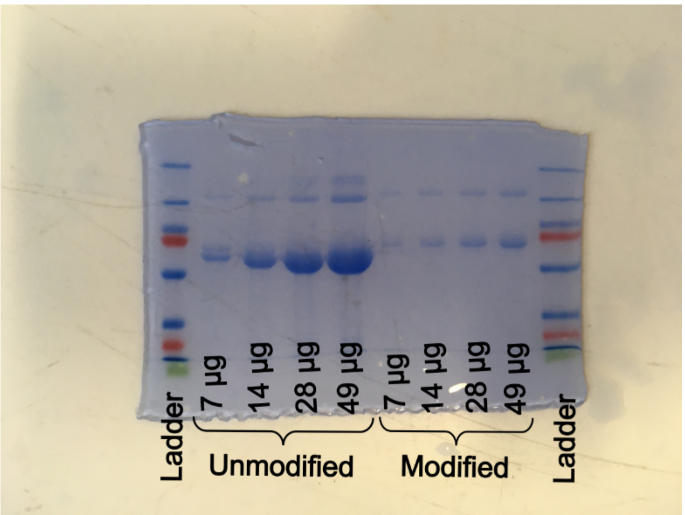

Figure S9. Uncropped SDS PAGE analysis of mannose-(Gly-Ser)$_3$-linked colicin Ia conjugate **9**

Lectin Blot

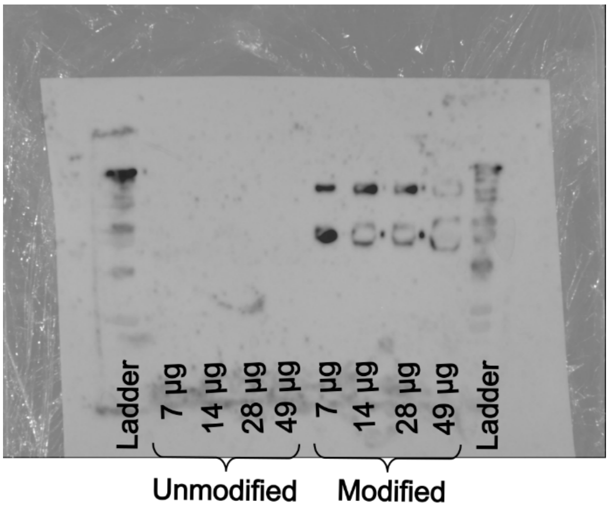

Figure S10. Uncropped Lectin blot analysis of mannose-(Gly-Ser)$_3$-linked colicin Ia conjugate **9**

Uncropped SDS-PAGE gel and Lectin blot

SDS-PAGE gel

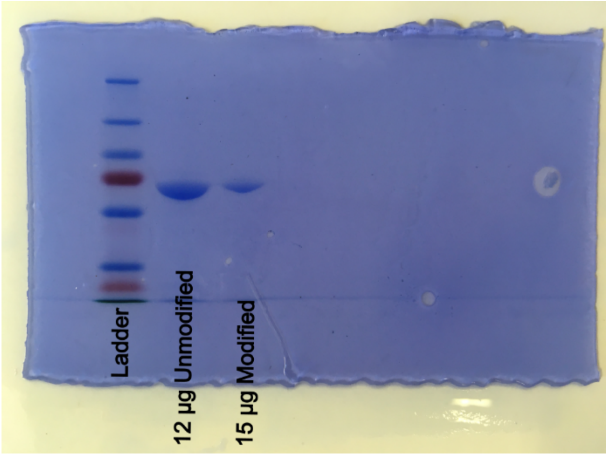

Figure S11. Uncropped SDS PAGE analysis of mannose-(Gly-Ser)$_6$-linked colicin Ia conjugate **10**

Lectin Blot

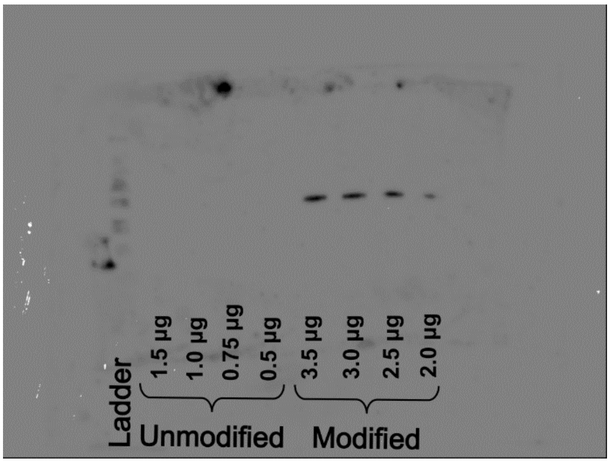

Figure S12. Uncropped Lectin blot analysis of mannose-(Gly-Ser)$_6$-linked colicin Ia conjugate **10**

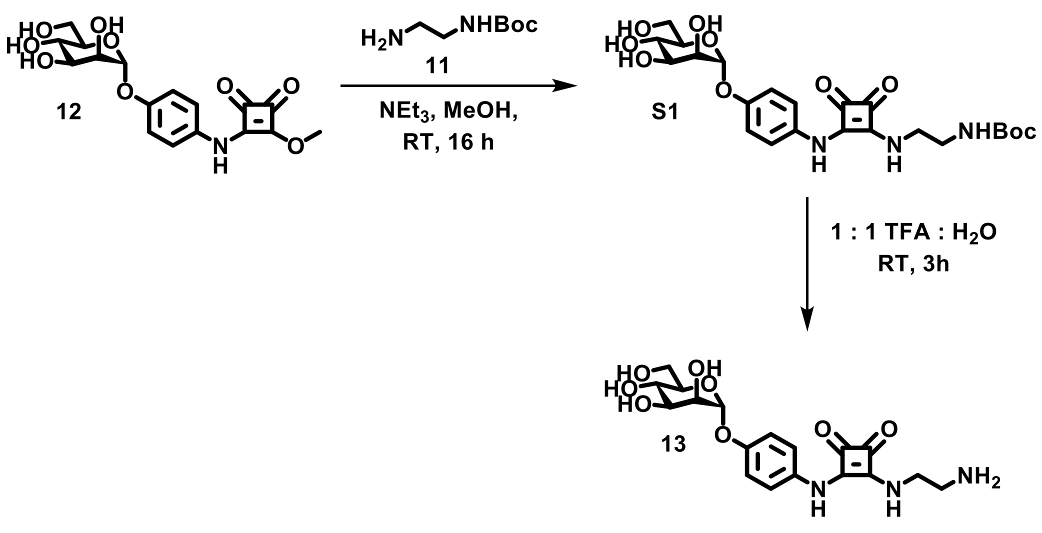

Scheme S3. Synthesis of mannosyl amine **13**

Hydrogen NMR Spectrum of Boc-protected amine mannose **S1**

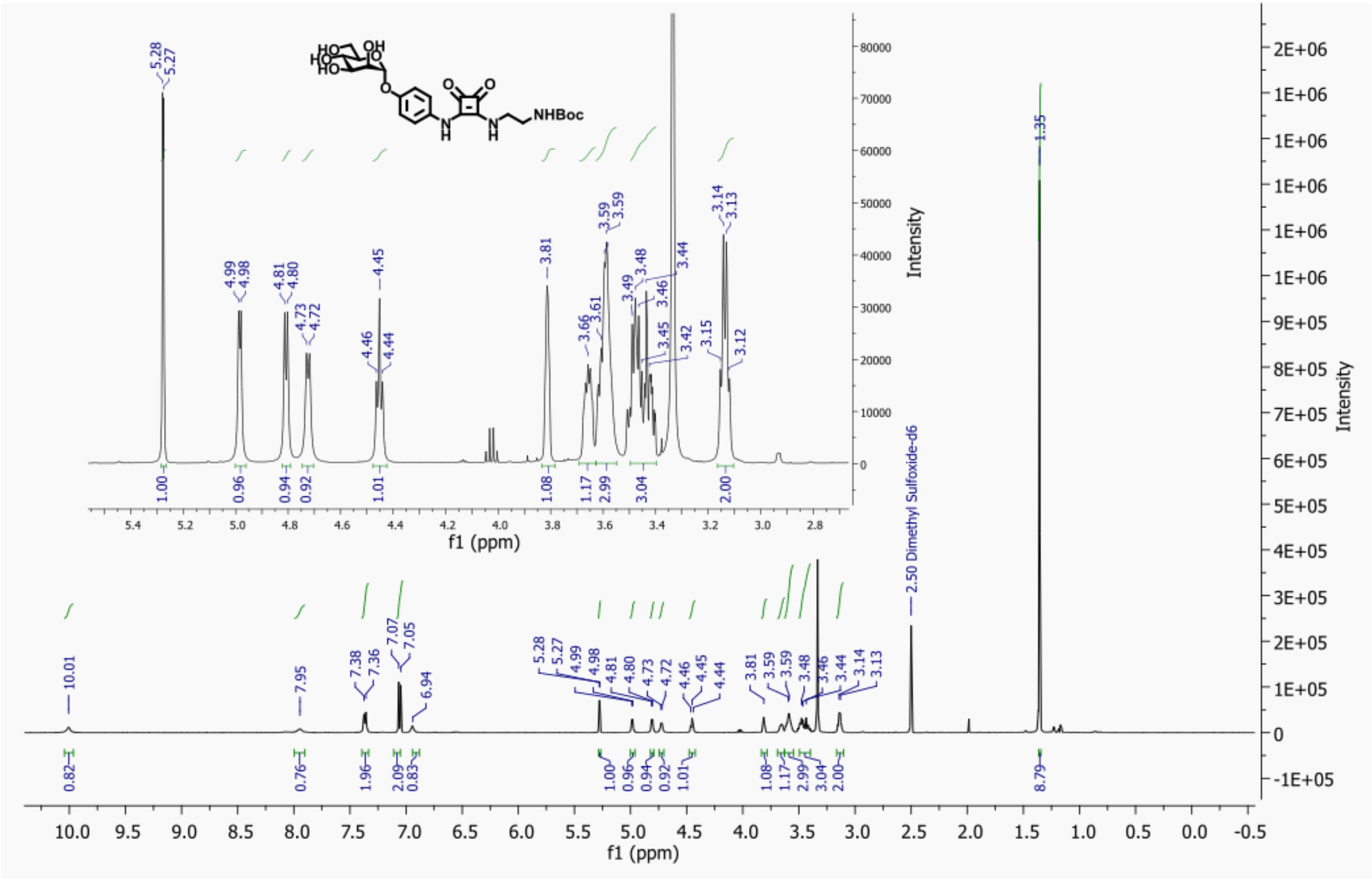

Figure S13. Hydrogen NMR of **S1**

Carbon NMR Spectrum of Boc-protected amine mannose **S1**

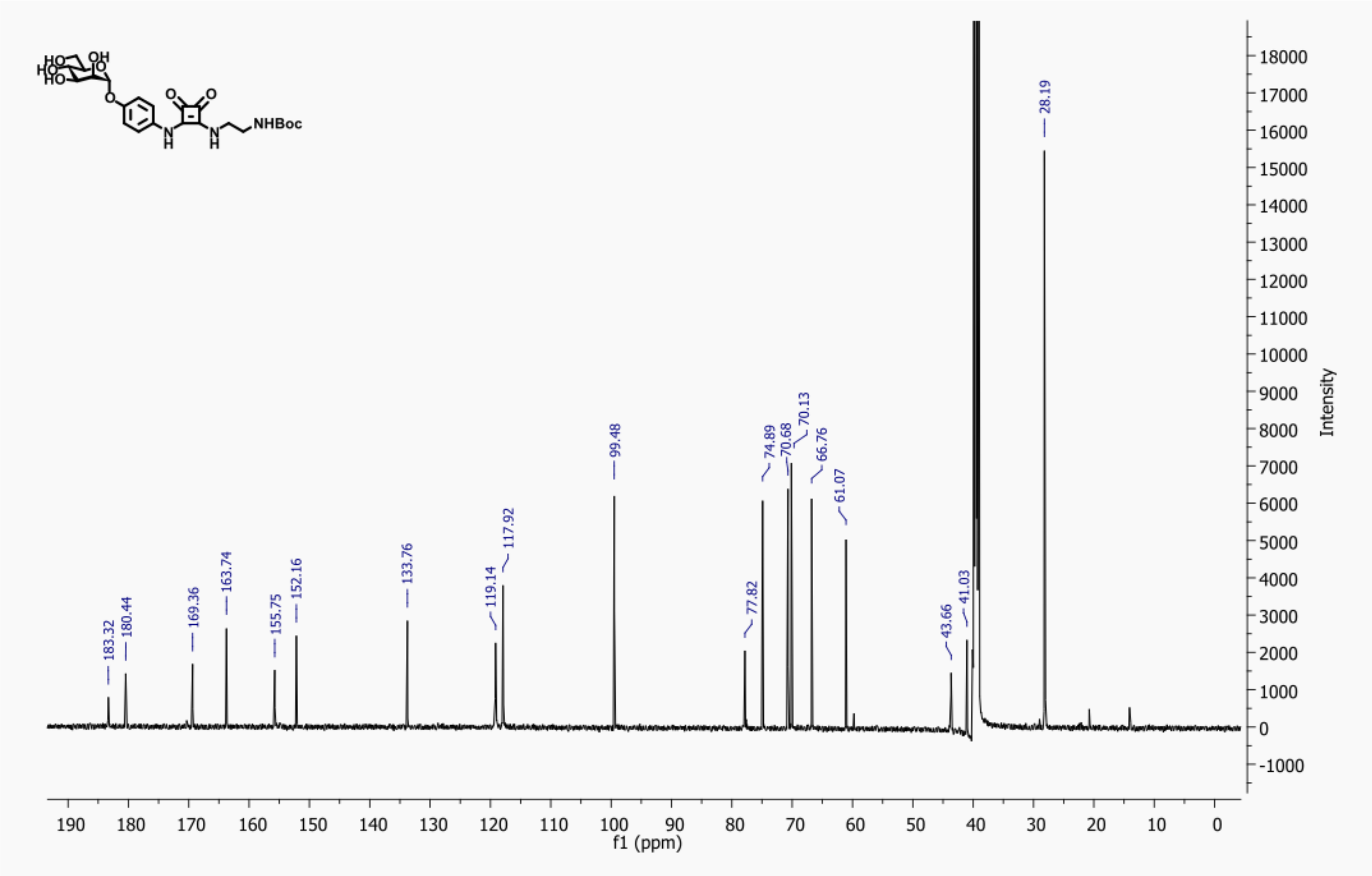

Figure S14. Carbon NMR of **S1**

Mass Spectrum of Boc-protected amine mannose **S1**

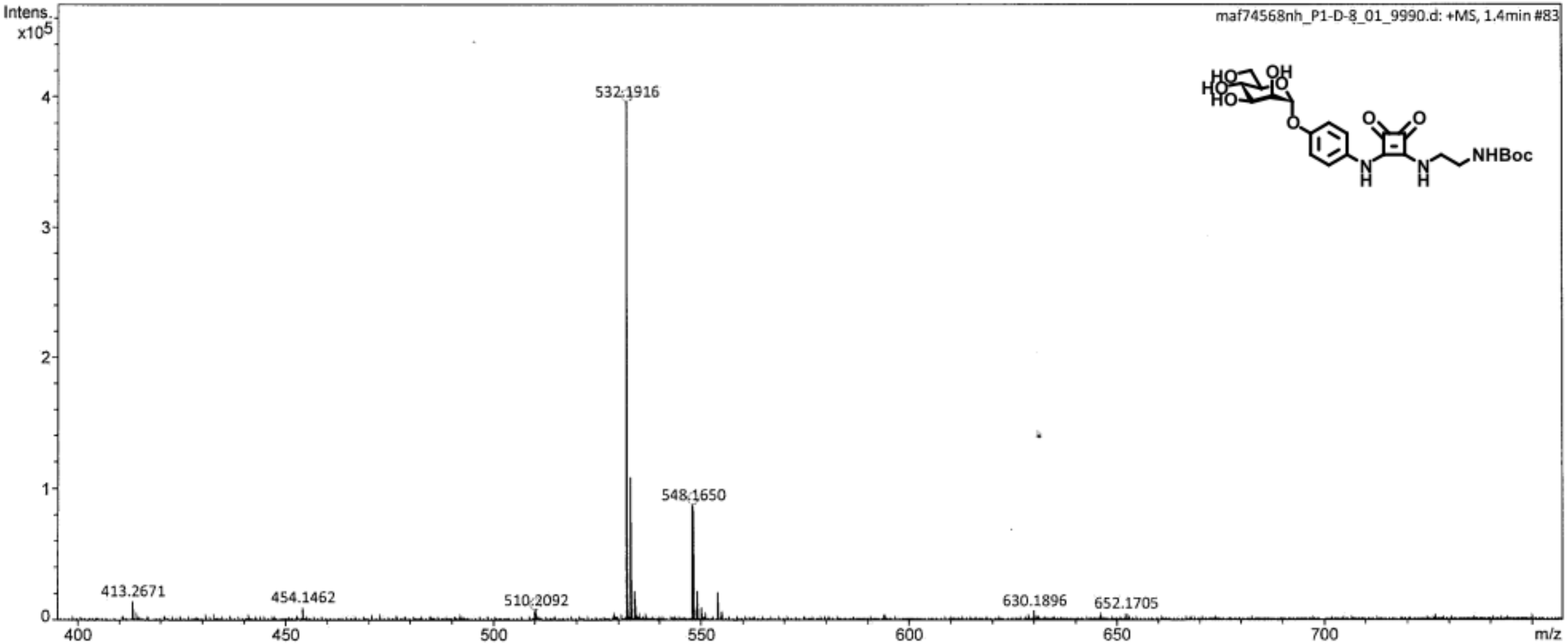

Meas. m/z	#	Ion Formula	m/z	err [ppm]	err [mDa]	mSigma	Mean err [ppm]
510.2092	1	C23H32N3O10	510.2082	-1.8	-0.9	14.7	-0.0
532.1916	1	C23H31N3NaO10	532.1902	-2.7	-1.4	4.0	-2.7
548.1650	1	C23H31KN3O10	548.1641	-1.7	-0.9	9.4	-0.7

Figure S15. Mass spectrum of **S1**

FT-IR (ATR) Spectrum of Boc-protected amine mannose **S1**

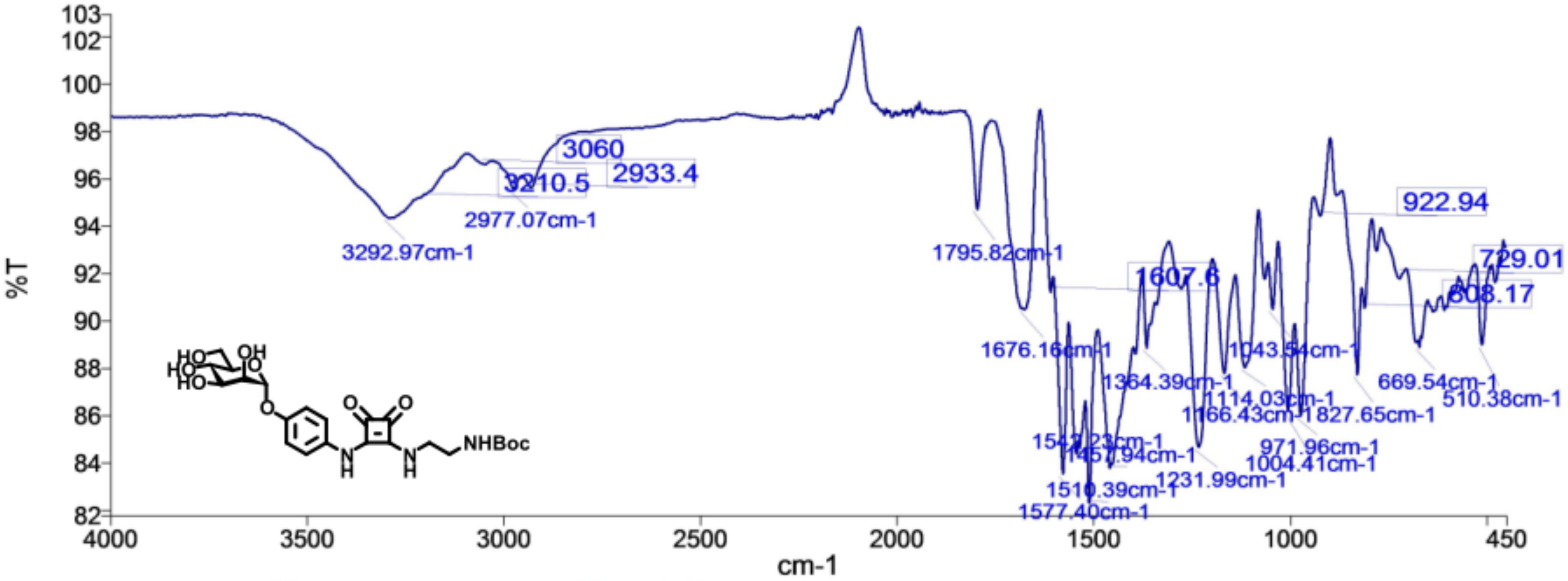

Figure S16. IR spectrum for **S1**

Hydrogen NMR Spectrum of amine-protected mannose **13**

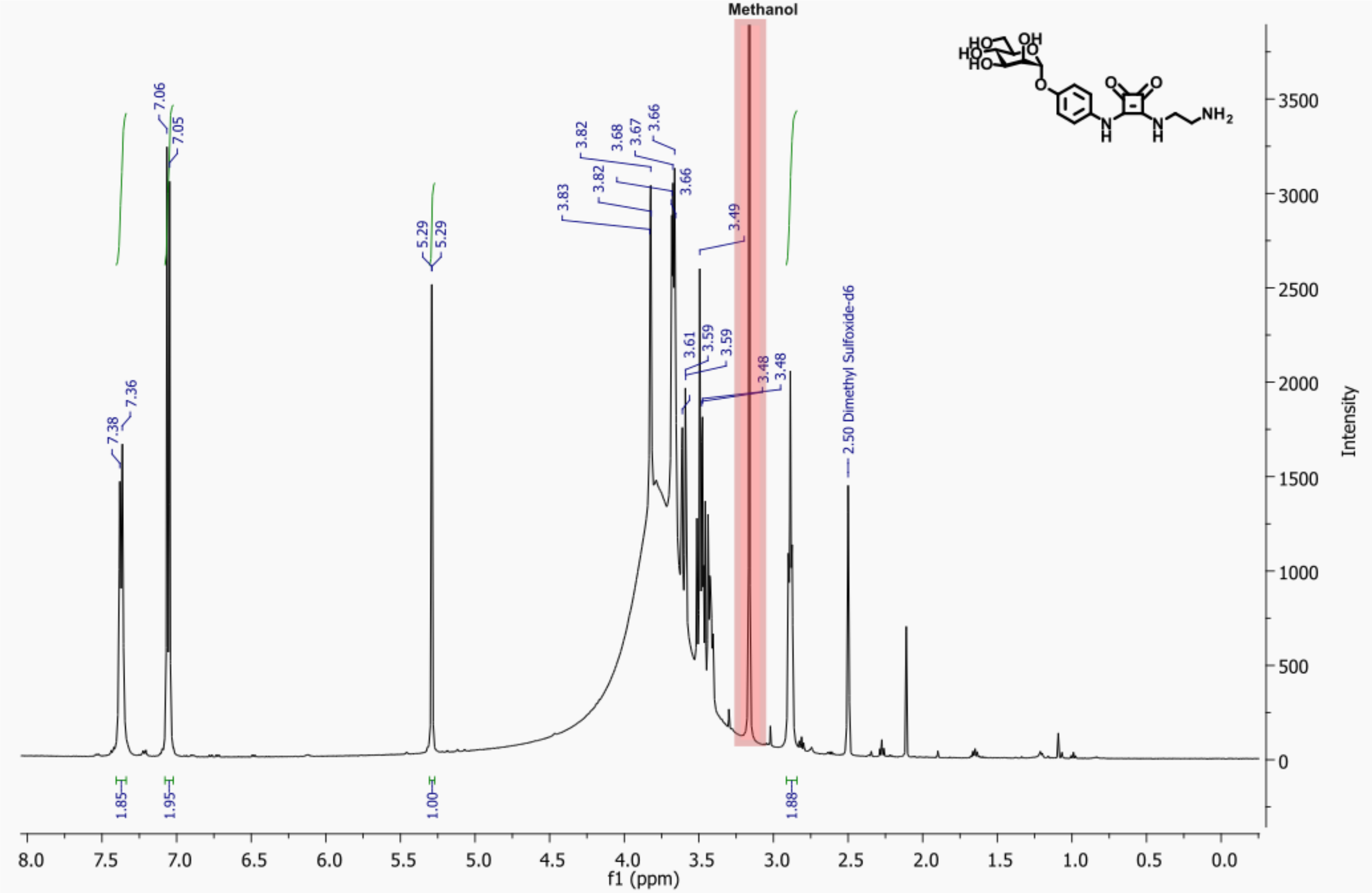

Figure S17. Hydrogen NMR of **13**

Carbon NMR Spectrum of amine-protected mannose **13**

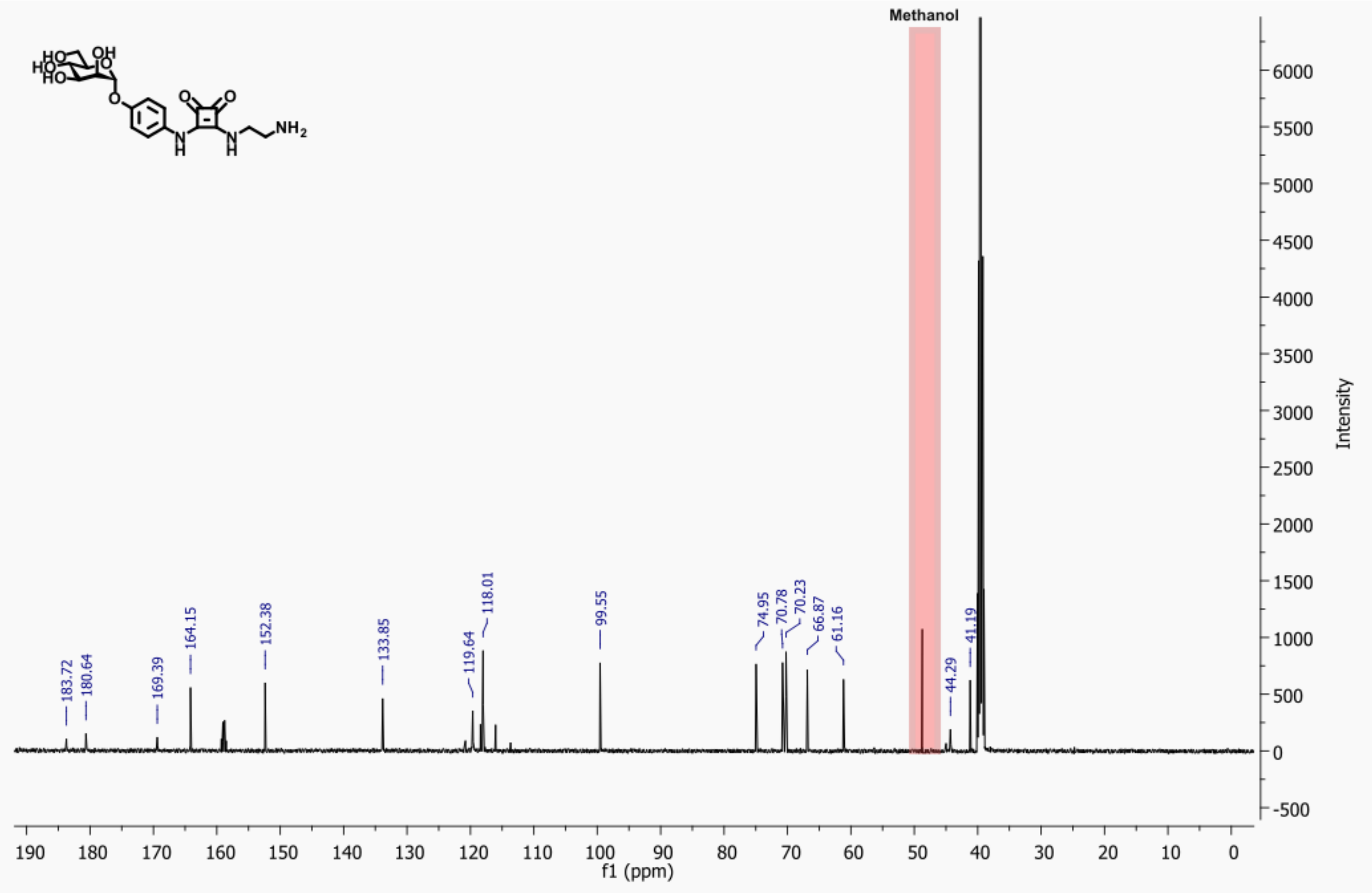

Figure S18. Carbon NMR of **13**

Mass Spectrum of amine-protected mannose **13**

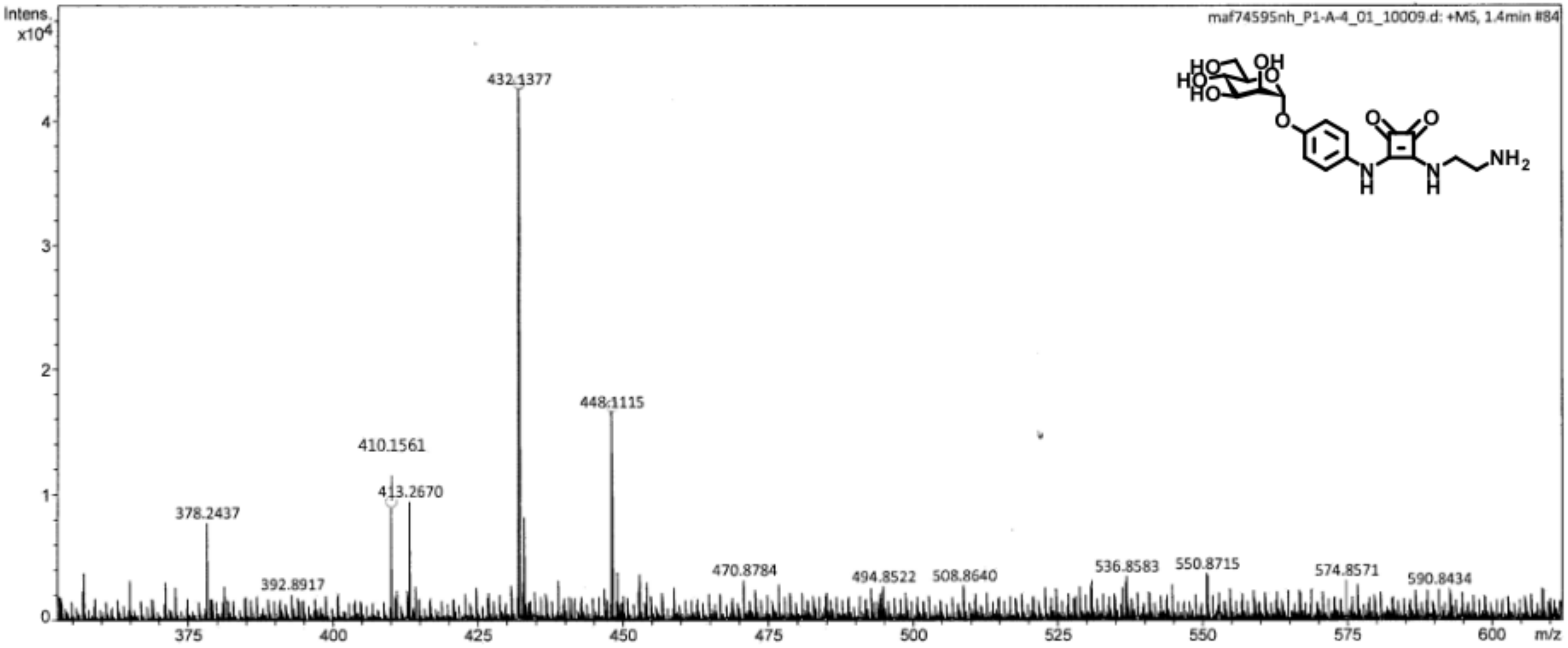

Meas. m/z	#	Ion Formula	m/z	err [ppm]	err [mDa]	mSigma	Mean err [ppm]
410.1561	1	C18H24N3O8	410.1558	-0.7	-0.3	28.6	0.3
432.1377	1	C18H23N3NaO8	432.1377	0.0	0.0	9.9	-1.2
448.1115	1	C18H23KN3O8	448.1117	0.4	0.2	10.5	-3.9

Figure S19. Mass spectrum of **13**

FT-IR (ATR) Spectrum of amine-protected mannose **13**

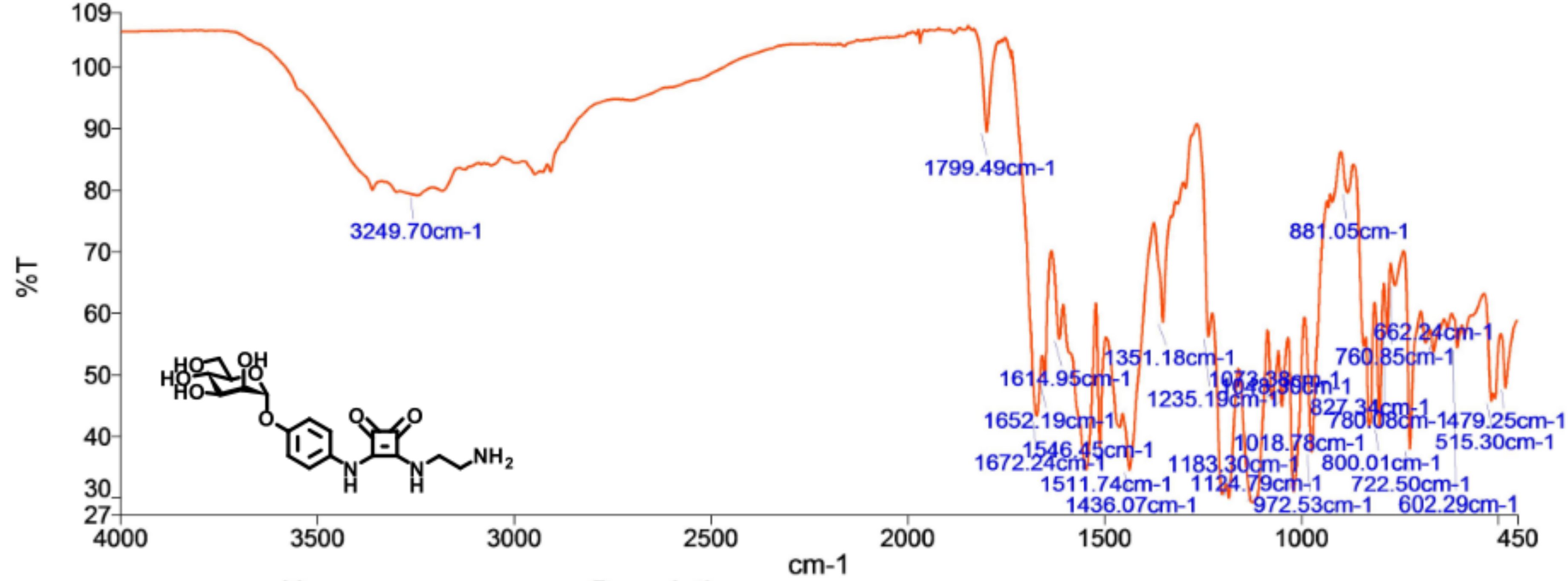

Figure S20. IR spectrum of **13**

Hydrogen NMR Spectrum of N-BOC-1,2-Diaminoethane **11**

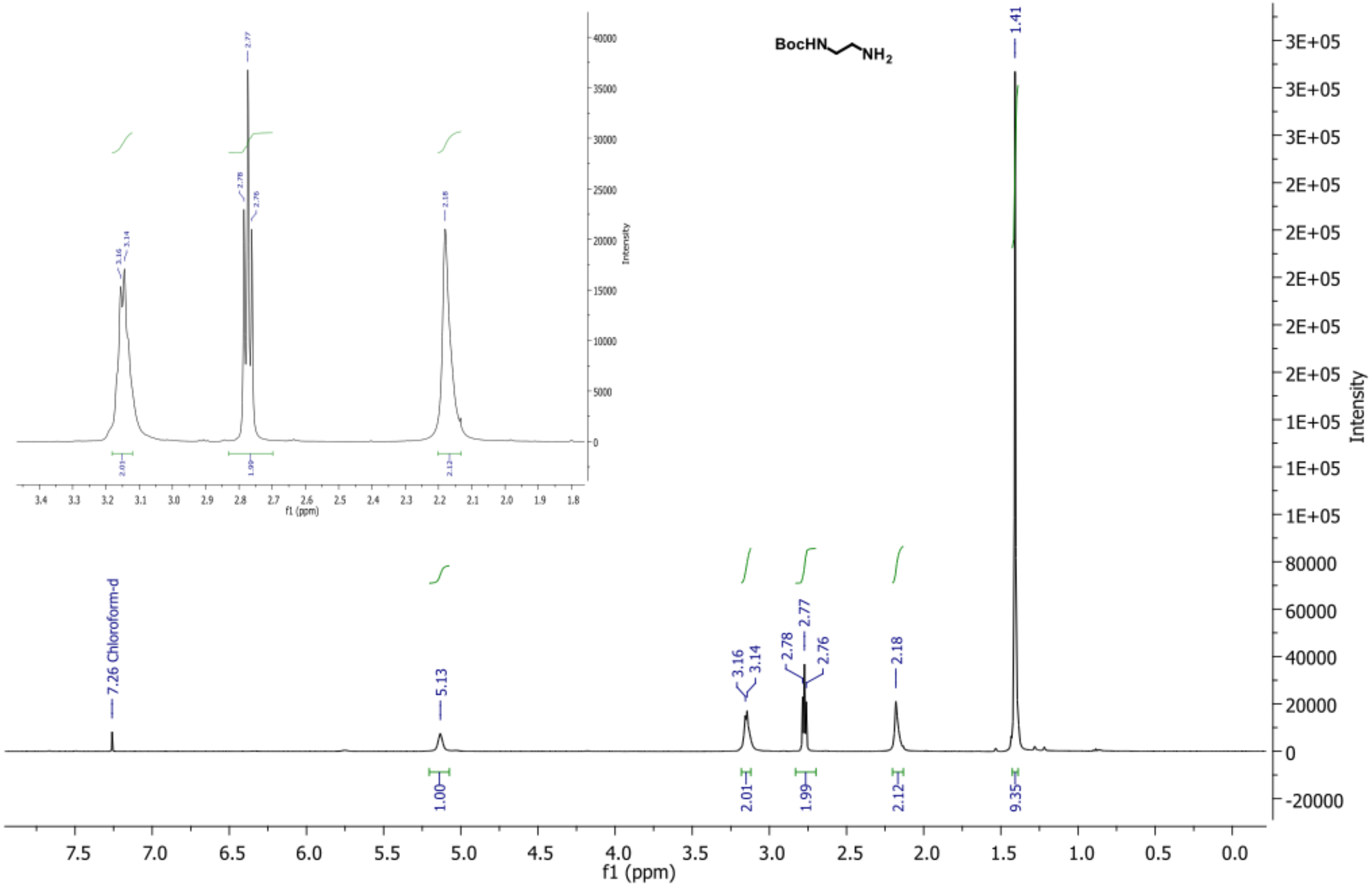

Figure S21. Hydrogen NMR spectrum of N-BOC-1,2-Diaminoethane **11**

Carbon NMR Spectrum of N-BOC-1,2-Diaminoethane **11**

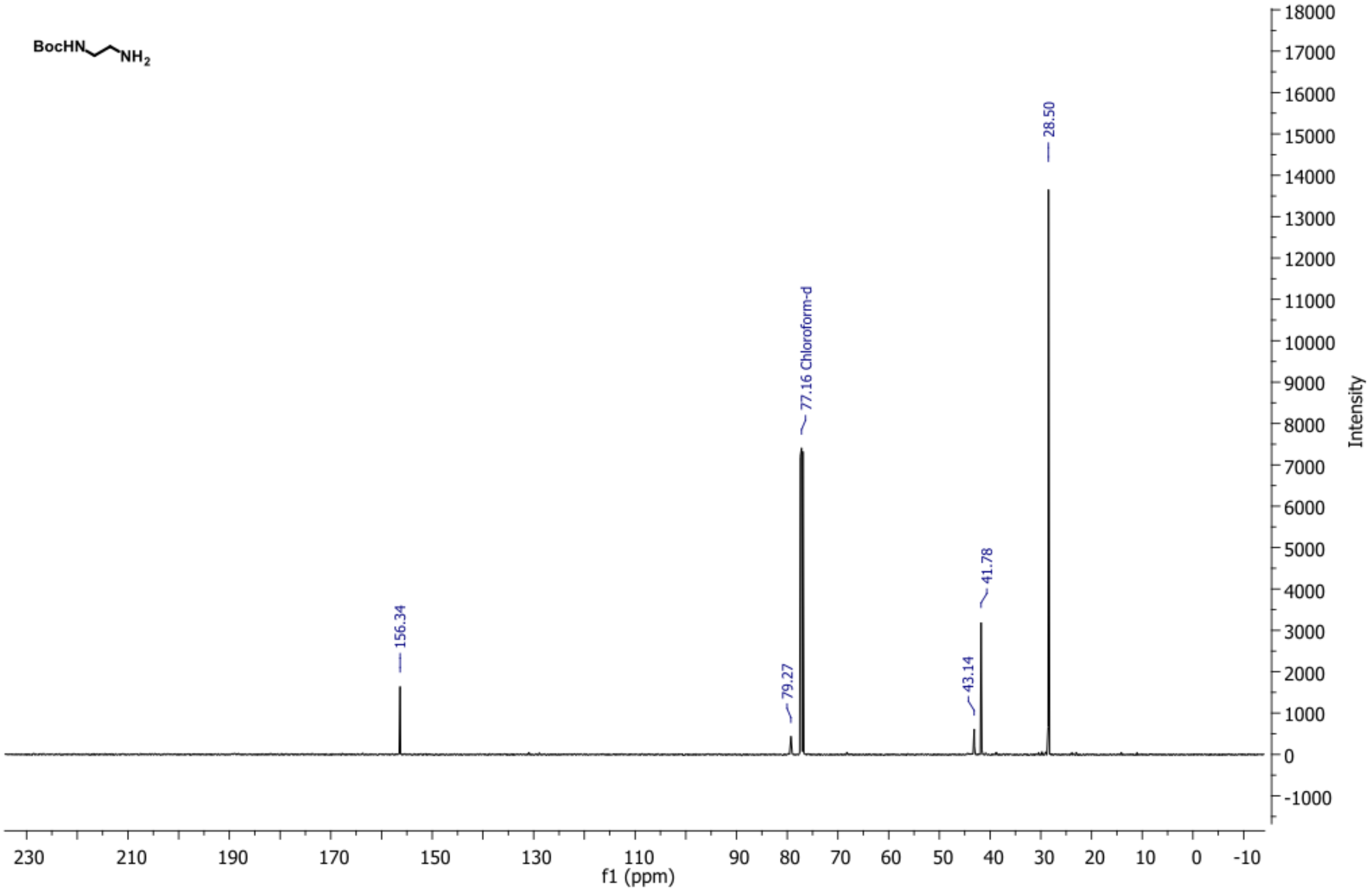

Figure S22. Carbon NMR spectrum of N-BOC-1,2-Diaminoethane **11**

Mass Spectrum of N-BOC-1,2-Diaminoethane **11**

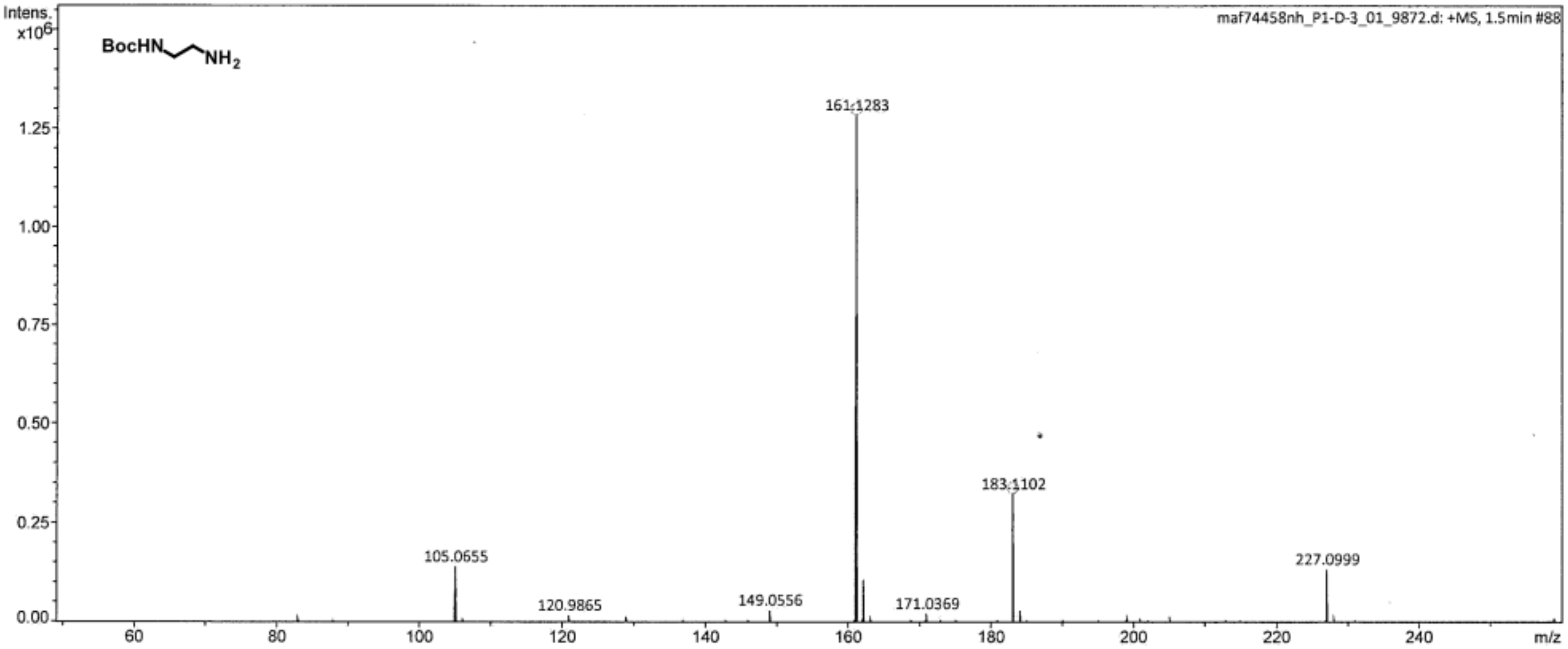

Meas. m/z	#	Ion Formula	m/z	err [ppm]	err [mDa]	mSigma	Mean err [ppm]
161.1283	1	C7H17N2O2	161.1285	0.9	0.1	1.8	0.4
183.1102	1	C7H16N2NaO2	183.1104	1.0	0.2	3.2	0.6

Figure S23. Mass spectrum of N-BOC-1,2-Diaminoethane **11**

FT-IR (ATR) Spectrum of N-BOC-1,2-Diaminoethane **11**

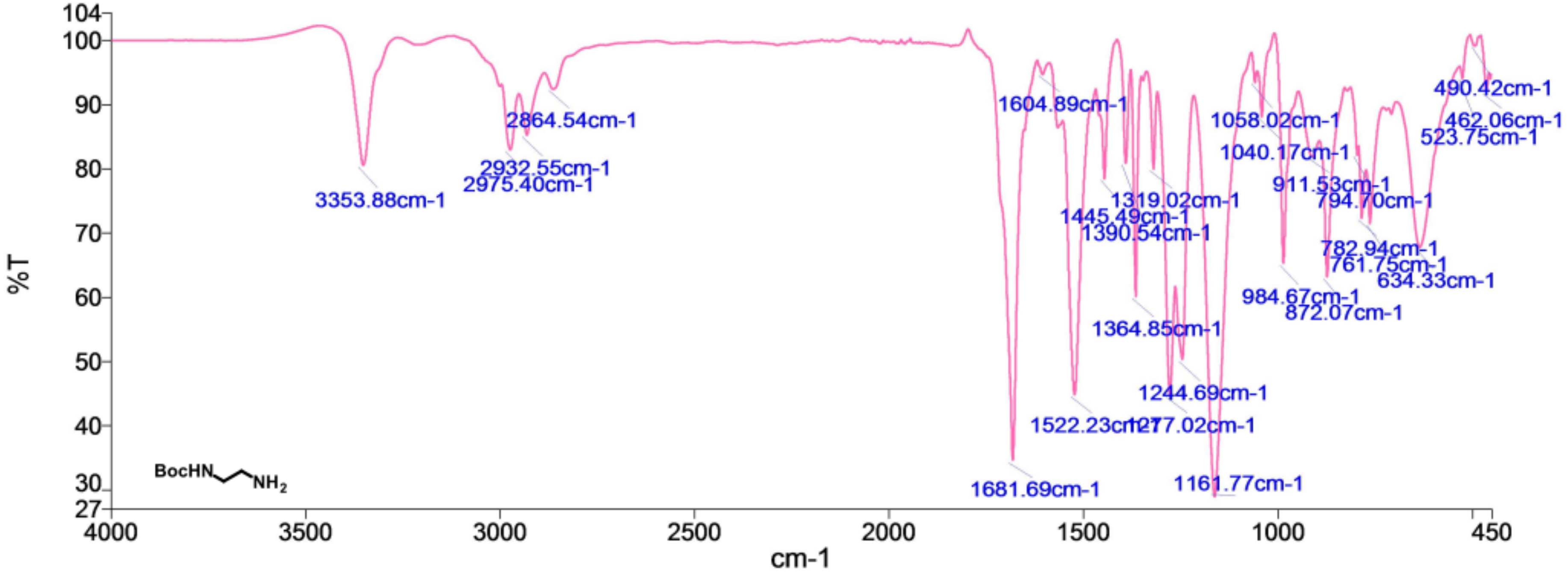

Figure S24. IR spectrum of N-BOC-1,2-Diaminoethane **11**

Formation of a mannose-linked colicin Ia conjugate using maleimide ligation

Methodology for the synthesis of a mannose-linked colicin Ia conjugate 16 using maleimide ligation

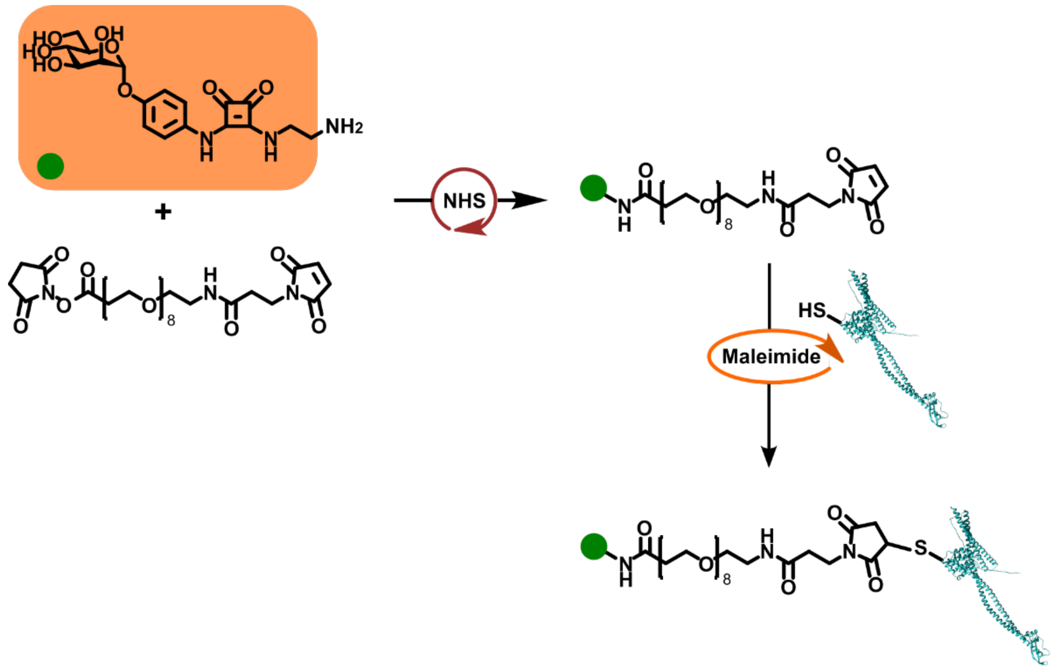

Scheme S4. Depiction of initial NHS ligation of mannose amine **13** to heterobifunctional linker **14**, followed by maleimide ligation to colicin Ia (PDB; 1CII[2])

Uncropped SDS-PAGE gel and Lectin blot

SDS-PAGE gel

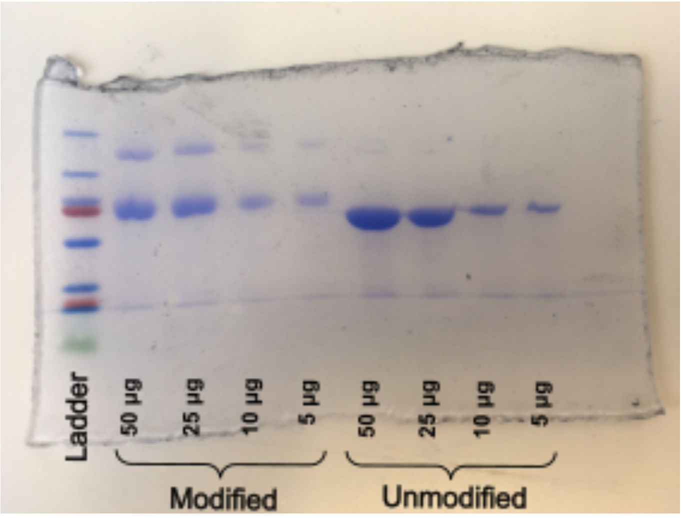

Figure S25. Uncropped SDS-PAGE analysis of mannose-maleimide-linked colicin Ia conjugate **16**

Lectin Blot

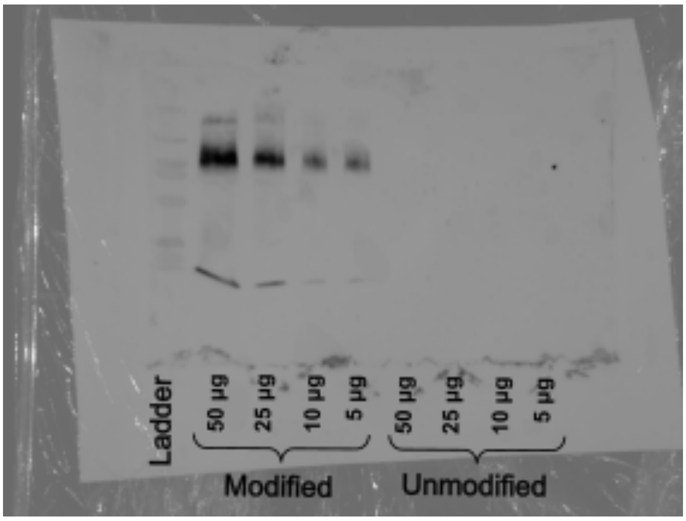

Figure S26. Uncropped Lectin blot analysis of mannose-maleimide-linked colicin Ia conjugate **16**

References

1. Rassam, P.; Copeland, N. A.; Birkholz, O.; Toth, C.; Chavent, M.; Duncan, A. L.; Cross, S. J.; Housden, N. G.; Kaminska, R.; Seger, U.; Quinn, D. M.; Garrod, T. J.; Sansom, M. S. P.; Piehler, J.; Baumann, C. G.; Kleanthous, C., Supramolecular assemblies underpin turnover of outer membrane proteins in bacteria. *Nature* **2015,** *523* (7560), 333-336.
2. Wiener, M. C.; Freymann, D. M.; Williams, P.; Ghosh, P.; Stroud, R. M., The crystal structure of colicin Ia. *Nature* **1997,** *72* (2), *385* (6615), 461-464.
